# Computer-Aided Solvent Selection for the Design of Integrated Synthesis, Crystallization, and Isolation Processes

**DOI:** 10.1021/acs.oprd.6c00168

**Published:** 2026-09-24

**Authors:** Mohamad H. Muhieddine, Suela Jonuzaj, Oliver Watson, Sara Ottoboni, Jan Sefcik, Chris J. Price, Shekhar K. Viswanath, Alan Armstrong, Amparo Galindo, George Jackson, Claire S. Adjiman

**Affiliations:** † Department of Chemical Engineering, The Sargent Centre for Process Systems Engineering, 4615 Imperial College London, South Kensington Campus, London SW7 2AZ, U.K.; ‡ Department of Chemical and Process Engineering, 3527 University of Strathclyde, Glasgow G1 1XQ, U.K.; § EPSRC Centre for Innovative Manufacturing in Continuous Manufacturing and Crystallisation, University of Strathclyde, Glasgow G1 1RD, U.K.; ∥ 1539 Lilly Research Laboratories, Indianapolis, Indiana 46082, United States; ⊥ Department of Chemistry and Institute for Molecular Science and Engineering, Imperial College London, Molecular Sciences Research Hub, White City Campus, Wood Lane, London W12 0BZ, U.K.

**Keywords:** CAM^*b*^D, synthesis, crystallization, isolation, mefenamic acid

## Abstract

The extensive use of solvents in pharmaceutical manufacturing has major impacts on overall process performance and final product quality. Solvent selection often requires tedious and resource-intensive experimental investigations. Nevertheless, the growing interest in embedding sustainable practices in process development workflows has motivated pharmaceutical manufacturers and researchers to deploy digital tools for enhancing process understanding and guiding the design of greener materials and processes. In this work, a computer-aided mixture/blend design (CAM^*b*^D) methodology is developed to identify simultaneously the optimal solvent or solvent/antisolvent mixtures, mixture compositions, and process conditions in end-to-end processes, considering integrated synthesis, crystallization, and isolation. Within the proposed approach, thermodynamic models are used to couple property prediction with process modeling to describe pharmaceutical process systems, while mathematical optimization techniques are applied to search the large design space of potential solvents and process conditions and identify the most promising designs among thousands of possible options. Further consideration is also given to the integrated design of reaction, crystallization, and wash solvents, where design decisions across the entire process are interlinked. The methodology is applied to the synthesis, crystallization, and isolation of mefenamic acid as a case study, while considering a broad range of process-wide key performance indicators (KPIs) that quantify product quality and resource efficiency, such as product purity and process energy consumption. In order to explore the trade-offs between competing KPIs, the proposed approach is extended to solve multiobjective design problems and generate a list of Pareto-optimal designs. The results highlight that the optimal designs are strongly dependent on the criteria considered and motivate the need for an integrated approach to solvent selection and process design. The findings of this study are expected to contribute to the digitalization of solvent selection for the accelerated development of sustainable pharmaceutical processes.

## Graphical abstract

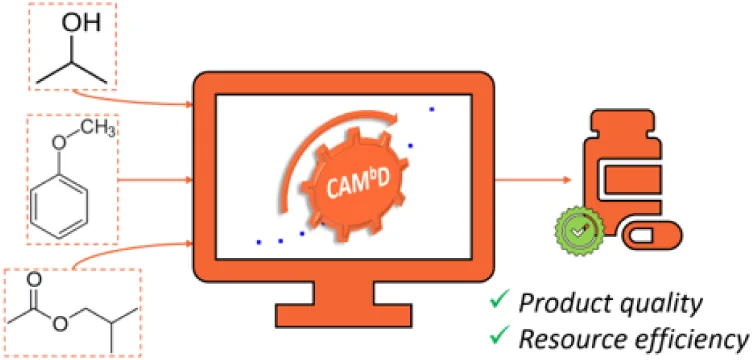

## Introduction

Solvents play a dominant role in the manufacturing of pharmaceutical products, as they are extensively used to accelerate synthetic reactions and to enable separation and purification.^1^ The production of active pharmaceutical ingredients (APIs) is a multi-step process involving several reaction and workup steps that consume large amounts of solvents, which significantly impacts process sustainability.^2–4^ This makes the pharmaceutical industry a wasteful chemical sector and highlights the need for systematic tools to enhance the resource efficiency of pharmaceutical processes.^5^

There has been growing interest in incorporating green chemistry principles into product design and development to enhance the sustainability of chemical manufacturing. In particular, solvent selection is currently regarded as an important research area within the chemistry and engineering communities, given the many solvent-related contributions to process performance metrics, including mass utilization, energy consumption, and process economics.^6^ This has motivated large pharmaceutical corporations such as Pfizer,^7^ Sanofi,^8^ and GSK^9,10^ to develop in-house solvent selection guides that include sustainability metrics and can guide the selection of high-performance solvents that pose minimal environmental and safety hazards. Additionally, a collaborative effort between the pharmaceutical industry and academia has led to the establishment of the CHEM21 solvent selection guide,^11^ which offers a preliminary sustainability assessment of solvents based on safety, health, and environment (SHE) criteria using physical properties and toxicity data.

Choosing the optimal solvents or solvent mixtures and the best operating conditions for a given pharmaceutical process can be challenging due to the discrete nature of the decisions (e.g., the number of solvents or the identities of the solvents). This can lead to a combinatorial explosion in the number of possible choices when a large solvent design space is considered. Furthermore, choosing a solvent or solvent mixture and process conditions often entails trade-offs between competing objectives; for example, enhanced product quality attributes can lead to increased cost and environmental impact. Currently, most practitioners choose solvents based on physico-chemical properties, environmental profiles, and solvent consumption levels, with the aim of reducing process costs and environmental footprint.^6,12^ Such a trial-and-error approach often requires performing expensive and time-consuming experimental investigations and likely identifies sub-optimal designs that fail to consider the integrated nature of all process decisions. Accordingly, efficient methodologies and more sophisticated tools that can highlight the most suitable designs from a holistic perspective are indispensable for the accelerated development of new pharmaceutical processes.

Computer-aided mixture/blend design (CAM^*b*^D)^13^ is a promising approach for identifying suitable solvents or solvent mixtures that meet predefined target properties and optimize a given performance measure. CAM^*b*^D has often been used in the design of optimal solvent mixtures for crystallization in order to facilitate and improve the production of pharmaceuticals. Karunanithi et al.^14,15^ proposed a decomposition-based approach for the design of binary solvent and/or antisolvent mixtures used in the crystallization of APIs, where smaller molecular and mixture design subproblems were posed and solved. The design of solvent mixtures for crystallization was also studied by Austin et al.,^16,17^ where the mixture design problem with a fixed number of ingredients was decomposed into two sequential optimizations: first, the choice of solvent components through the design of molecular structures and then the optimization of the mixture composition (mole fractions). Jonuzaj et al.^18,19^ presented a general CAM^*b*^D formulation based on generalized disjunctive programming (GDP)^20^ for the selection and design of solvent mixtures for separation processes in which the number of ingredients in the blend as well as the identities of the solvents and their mole fractions are optimized simultaneously. Beyond CAM^*b*^D approaches, the literature includes numerous examples of modeling pharmaceutical manufacturing processes and evaluating them from a technoeconomic perspective, particularly for batch and continuous crystallizations, covering APIs such as ampicillin,^21^ cephalexin,^22^ flurbiprofen,^23^ rufinamide,^24^ and cyclosporine, paracetamol, and aliskiren.^25^

Despite the aforementioned advances, most solvent design or selection approaches suffer from two main limitations. First, solvents are often designed or selected based on fixed operating conditions; this leads to identifying sub-optimal designs, as it significantly reduces the operating space being considered and potentially overlooks promising designs. Second, most of these approaches are focused on optimizing a single process unit rather than taking the full integrated process into account. Selecting optimal solvents for a specific unit (e.g., crystallization) without consideration of the overall process may negatively impact the effectiveness of subsequent process steps, and thus reduce the efficiency of the overall process. Recently, the limitation of fixed operating conditions has been addressed. For example, Watson et al.^26,27^ proposed a solvent mixture design formulation for the hybrid cooling and antisolvent crystallization of APIs where the initial and final crystallization temperatures are treated as decision variables and relevant thermodynamic properties are calculated using the SAFT-*γ* Mie equation of state.^28^ A similar problem was recently addressed using a model derived from machine learning to predict solvent-dependent thermodynamic properties.^29^ The limitations of taking a sequential, unit-by-unit approach to design have also been addressed in research studies focused on solvent selection for integrated pharmaceutical processes. Wang and Lakerveld^30^ presented a systematic approach to identify the optimal solvents and process conditions for continuous antisolvent crystallization with solvent recycling via single-stage flash separation. The authors used the perturbed-chain statistical associating fluid theory (PC-SAFT) model^31,32^ to predict relevant thermodynamic properties (solubility and vapor-liquid equilibrium) and the continuous molecular targeting (CoMT) approach^33^ to solve the optimization problem efficiently. The same research group extended their approach to consider different process topologies, such as continuous crystallization with antisolvent separation and recycling by multi-stage distillation,^34^ reactive crystallization with raw material recycling,^35^ and continuous synthesis, liquid–liquid extraction, and crystallization systems.^36^ However, a main limitation of the CoMT approach is that it does not lead to a fully integrated design due to the disconnection between pure component parameters and actual molecules. Jonuzaj et al.^37^ developed a comprehensive CAM^*b*^D-based approach for integrated crystallization and isolation processes that considers a combined cooling and antisolvent crystallization process where optimal solvent and antisolvent mixtures, their proportions in solution, and optimal process conditions were determined at the same time, while maximizing crystal yield and reducing solvent consumption. In this approach, the UNIFAC model^38^ was used to predict mixture thermodynamics, and critical interlinked design decisions, such as API solubility across both crystallization and isolation stages, as well as the miscibility of crystallization and wash solvents, were taken into account. Furthermore, environmental, health, and safety performance measures were considered in the comprehensive model to prioritize safe and environmentally benign solvents. Muhieddine et al.^39^ proposed a CAM^*b*^D-based solvent selection tool for integrated synthesis and crystallization processes to identify simultaneously the optimal solvent or solvent/antisolvent mixtures, mixture compositions, and process conditions that optimize green chemistry and product quality metrics, such as the solvent E-factor (SEF) and crystal yield, respectively. In this study, different objective functions and design spaces were considered, the UNIFAC model was used to predict mixture thermodynamics, and the benefits of implementing an integrated approach to solvent selection as opposed to a sequential approach were highlighted. Chai et al.^40^ applied the Grand Product Design framework^41^ to select and design solvents for crystallization and isolation (filtration, washing, and drying), and included several sub-models that can accommodate multiple performance criteria, including environmental impact. Ottoboni et al.^42^ developed a workflow for solvent selection for crystallization and isolation that combines experimental and modeling approaches. In their workflow, crystallization solvents are first selected based on their performance in terms of maximizing crystal yield and minimizing solvent consumption. Then, suitable wash solvents are identified based on their safety, washing efficiency (i.e., maximizing impurity removal and minimizing API dissolution), and compatibility with the selected crystallization solvents (e.g., similar physical properties) and with the drying step (e.g., low-boiling-point wash solvents). Isolation performance was predicted by simulating the material balances of the isolation steps of filtration and washing, and quantifying important indicators such as impurity removal, wash solvent consumption, and the solubility behavior of APIs and impurities during washing.

The aim of the current work is to further contribute to the digitalization of the solvent selection task during early-stage pharmaceutical process development by considering integrated process decisions across end-to-end drug substance manufacturing. Accordingly, in this paper and building on the work of Jonuzaj et al.^37^ and Muhieddine et al.,^39^ a CAM^*b*^D-based approach to solvent selection and process design for integrated synthesis, crystallization, and isolation (filtration, washing, and drying) is proposed, with a focus on metrics that quantify product quality and material/energy efficiency. To the best of the authors’ knowledge, a CAM^*b*^D-based approach for guiding solvent selection and process design across end-to-end drug substance manufacturing, while considering multiple key performance indicators (KPIs) of process performance, has not previously been reported. Similar to the thermodynamics-based approach of Muhieddine et al.,^39^ the proposed design tool focuses on the thermodynamic behavior of a target pharmaceutical compound and its impurities in solution across multiple process operations to shortlist promising design options (solvent identities + process conditions); these options can then be taken forward to the next steps of process development and assessed based on additional criteria such as kinetics, transport phenomena, and crystal shape. The proposed framework can be used to rank solvent mixtures using a process model based on equilibrium thermodynamics. In practice, however, kinetic and transport effects can impact both predicted process performance and the resulting solvent order. Solvent-specific reaction rates and selectivity may shift the optimal operating window, affecting achievable conversion and impurity formation. Similarly, crystallization is often controlled by nucleation and growth kinetics and by mixing-dependent supersaturation; thus, solvents with comparable equilibrium solubility can still produce different particle attributes, filtration behavior, and cycle times, with knock-on effects on productivity and cost. Transport properties (e.g., viscosity, diffusivity, density, and interfacial tension) further influence mixing, phase separation, and mass/heat transfer, potentially introducing rate limitations not captured by an equilibrium model. Accordingly, the solvent/process design ranking obtained from the presented modeling approach should be treated as an equilibrium-based feasibility and performance screen that can inform experimental campaigns by eliminating solvent options that lead to poor performance and prioritizing promising choices. Final solvent selection should then be confirmed using kinetics and transport data, particularly when reaction or crystallization is rate-limiting. As a demonstration of the presented modeling approach, the method is applied to the synthesis, crystallization, and isolation of mefenamic acid, resulting in a shortlist of high-performance solvent candidates.

The paper is structured as follows: first, the CAM^*b*^D problem statement and mathematical formulation are presented, including a detailed discussion of the process model and design constraints for the synthesis, crystallization, and isolation steps, as well as the process-wide KPIs of interest. Then, the proposed methodology is applied to identify optimal solvent and process designs for the end-to-end production of mefenamic acid, considering different objective functions and solving both single-objective and multiobjective design problems. Finally, conclusions on the proposed design approach and its applicability, as well as a statement on future work, are provided.

## Problem Definition and Formulation

### General Design Problem Formulation

The proposed CAM^*b*^D problem can be described as follows: Given a synthetic step in the manufacturing of a pharmaceutical compound and a specified production rate, reaction conversion and selectivity, product purity, and a list of possible solvents, identify a ranked list of candidate solvent mixtures as described by the identities of the solvents, mixture compositions, and process conditions for the corresponding integrated synthesis, crystallization, and isolation (filtration, washing, and drying) process that optimize one or more KPIs. In the current formulation, KPIs that describe process-wide performance, rather than KPIs that are limited to a single process unit, are considered.

An optimization-based framework is proposed, as it allows for the efficient consideration of the interplay between solvent identities and the optimal process variables—e.g., the maximum temperature that can be used depends on the bubble point of the solvent mixture. Through the use of optimization, the identification of promising solvent combinations is based on an assessment of the best performance (KPIs) that each combination could deliver. Conversely, each solvent combination that is not selected is discarded on the basis that the best performance that could be achieved with that choice is comparatively poor. Finally, an optimization formulation provides a readily extensible platform to handle future developments that may result in increases in problem complexity, such as the number of discrete combinations of solvents (here, several thousand are considered), the number of continuous variables, and equations that may arise from the addition of units to the process flowsheet or from the use of more detailed models.

Before presenting the formulation, a generic end-to-end pharmaceutical process step is shown in Figure 1 to illustrate possible decision variables. This process configuration can apply to a batch or continuous process; in the case of a batch process, each stream represents the initial or final point of the batch operation. Adopting the language of continuous processes without loss of generality, the process can be described as follows. Stream 1, the feed stream, is made up of reactants and impurities dissolved in either a pure solvent *s*1 or a binary solvent mixture (*s*1+*s*2) that enters a heat exchanger at a temperature *T*_1_ (K). The heated reaction mixture enters the reactor as stream 2 at a temperature *T*_2_ (K), where the reaction proceeds in a single step and can be accompanied by competing unwanted/side reactions. The product stream exiting the reactor, stream 3, is a liquid mixture of the desired product **D**, unreacted material, impurities, and unwanted side and byproducts at temperature *T*_3_ (K). This homogeneous stream is sent to a crystallizer, which can be designed as a cooling, antisolvent, or hybrid cooling and antisolvent crystallization unit. In the case of antisolvent crystallization, stream 4, composed of pure solvent *s*2 at temperature *T*_3_, is fed to the crystallizer. The crystallizer outlet is a slurry composed of a liquid phase, stream 5, normally containing dissolved amounts of all process components, and a solid phase, stream 6, typically a pure solid form of **D**. Both streams 5 and 6 are at temperature *T*_5_ (K). After crystallization, the slurry that exits the crystallizer is sent to a filtration unit, where solvents and impurities are removed in the filtrate stream, stream 7, leaving behind a wet filter cake consisting of a liquid phase of solvents/dissolved impurities trapped inside the pore volume of the cake, stream 8, and a solid phase of crystallized API, stream 9. The wet filter cake is then sent to a washing unit, where it is washed with stream 10, which consists of pure wash solvent, denoted by *sw*, to remove any excess impurities and increase product purity. Stream 11 represents the filtrate stream that exits the washing unit, while streams 12 and 13 represent the liquid and solid phases of the filter cake obtained after the washing, respectively. Finally, the wet filter cake from the washing unit is sent to drying, where the solvents are evaporated to form vapor stream 14, and a dry pharmaceutical compound is produced as stream 15. We note that in the process flowsheet as described, any antisolvent used for crystallization must be the same as solvent *s*2 in stream 1. This makes it possible to exploit the solubility maximum often seen in solvent mixtures without adding an additional solvent to the process. Furthermore, the wash solvent is taken as prespecified, so that the focus for the washing unit is on ensuring that solvents *s*1 and *s*2 are compatible with the specified wash solvent. It is, of course, possible to lift these restrictions and allow different solvents to be used for crystallization and washing, but this is not considered in this work.

**Figure 1:**
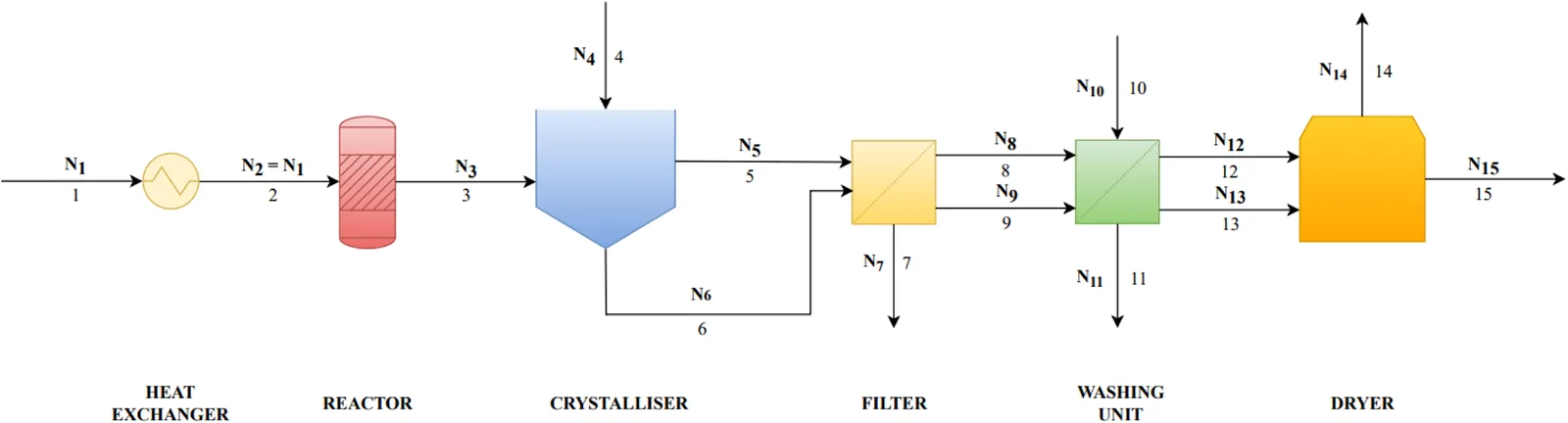
The conceptual flowsheet used for the solvent selection problem of the integrated synthesis, crystallization, and isolation process. **N** denotes the vector of component mole numbers (batch process) or component molar flow rates (continuous process).

Among the many criteria that must be considered when quantifying solvent effects on process performance,^43^ the proposed CAM^*b*^D approach to solvent selection focuses on thermodynamic driving forces, such as the solubility of reactants, impurities, and products, in order to shortlist a set of solvent candidates. These candidates can then be evaluated based on other considerations, such as reaction/crystallization kinetics, transport properties, and particle size distribution. Importantly, the CAM^*b*^D formulation accounts for the impact of interlinked design decisions in the end-to-end process; for example, the choice of solvents *s*1 and *s*2 for dissolving the reaction species in the reaction step must be compatible with the crystallization step, so that a high yield of solute **D** and effective impurity rejection can be achieved, and with the washing step, by being miscible with the wash solvent *sw* and further rejecting impurities (and not dissolving **D**). Such specifications are crucial for ensuring the design of feasible and efficient end-to-end drug-substance manufacturing processes.

It is useful to assign different definitions to the terms “component” and “species” used in describing the design formulation; here, “component” denotes a constituent of a process stream, i.e., a reactant, an impurity, a product, solvent *s*1 or solvent *s*2, whereas “species” denotes a molecule in the list of solvents from which *s*1 and *s*2 are selected. Furthermore, in order to develop a generic formulation of the design problem, a number of sets are defined:

- The set *C* comprises all the components that appear in at least one of the liquid streams;
- the set *C* ^*K*^ comprises all the components that may appear in solid form in the process, i.e., whose melting point is within the range of process operating temperatures. In principle, this may introduce new components if co-crystals or solvates can form. However, here it is assumed that only pure crystal forms can be produced so that *C* ^*K*^ ⊂ *C*;
- The set *J* comprises all solid components that may appear as impurities in the final dried product, i.e., all components in set *C* ^K^ except the desired product;
- The set *Q* comprises solvents *s*1 and *s*2 that are selected for the integrated process (*Q* = {*s*1, *s*2});
- The set *V* comprises all the solvents in the process, including the set *Q* and the wash solvent *sw* (*V* ={*s*1, *s*2, *sw*});
- The set *S* comprises the solvent candidates from which solvents *s*1 and *s*2 are chosen and thus must contain at least two elements;
- The set *R* comprises indices/names for all of the reactions;
- The set *T* consists of the indices for all the process streams introduced earlier and shown in Figure 1.

The CAM^*b*^D formulation can be developed using Generalized Disjunctive Programming (GDP)^20^ and then expressed as a MINLP using the big-M approach.^44^ The mathematical structure of the multiobjective optimization (MOO) version of the problem can be represented as follows:

1 $$\begin{array}{ll} \underset{x,y}{\min} & f_{1}\left(x\right),...,f_{P}\left(x\right)\\ \mathrm{s.t.} & h_{0}\left(x\right)=0\\ & g_{0}\left(x\right)\leq 0\\ & g_{i,s}\left(x\right)\leq M_{i,s}\left(1-y_{i,s}\right),i\in \left\{s1,s2\right\};s\in S\\ & Ay\leq a\\ & x\in \left[x^{L},x^{U}\right]\subset R^{n}\\ & y\in \{0,1{\}}^{u} \end{array}$$

where each objective function *f*_*p*_(**x**), *p* = 1,···,*P* represents a KPI that describes process-wide performance, such as process yield or product purity; **x** is an *n*-dimensional vector of continuous variables, such as the number of moles, process temperatures, and mixture compositions; and **y** is a *u*-dimensional vector of binary variables representing solvent identities. This includes the variables *y*_*i,s*_, *i* ∈ *Q*, and *s* ∈ *S*, expressed as

$$y_{i,s}=\{\begin{array}{ll} 1, & \mathrm{if solvent species},s,\mathrm{is assigned to solvent component}\;i,\\ 0, & \mathrm{otherwise.} \end{array}$$ 2

The vector **h**_0_(**x**) represents process model constraints; the vector **g**_0_(**x**) represents design specifications that are independent of the discrete choices; the vector **g**_*i,s*_(**x**) represents design and operating specifications that depend on solvent identity; **M**_*i,s*_ is a vector of positive or negative big-M values; and **A** and **a** are a constant matrix and vector, respectively, used to describe the logical relationships between the binary variables. Vectors **g**_0_(**x**) and **g**_*i,s*_(**x**) include design and operating specifications such as API solubility across synthesis, crystallization, and isolation, as well as the miscibility of *s*1 and *s*2 with *sw*.

For design problems in which two KPIs are optimized (*P* = 2), the *ϵ*-constraint method^45^ is used to solve the multiobjective CAM^*b*^D problem, while for design problems in which three KPIs are optimized (*P* = 3), the augmented *ϵ*-constraint method (AUGMENCON)^46^ is used in order to guarantee the Pareto optimality of the generated solutions. AUGMENCON involves the use of slack or surplus variables to transform the objective-function *ϵ*-constraints to equality constraints and the use of these variables as a second term in the main objective function with lower priority. The reader is referred to Mavrotas^46^ for a proof that AUGMENCON produces only efficient or non-dominated solutions. The general mathematical formulation of AUGMENCON, assuming without loss of generality that the multiple objective functions are to be maximized, is given by

3 $$\begin{array}{ll} \underset{x,y}{\max} & f_{1}\left(x,y\right)+\kappa \left(\overset{P}{\underset{p=2}{\sum }}\frac{s_{p}}{r_{p}}\right)\\ \mathrm{s.t.} & f_{p}\left(x,y\right)-s_{p}=\epsilon_{p},\;p=2,...,P\\ & h_{0}\left(x\right)=0\\ & g_{0}\left(x\right)\leq 0\\ & g_{i,s}\left(x\right)\leq M_{i,s}\left(1-y_{i,s}\right),i\in \left\{s1,s2\right\};s\in S\\ & Ay\leq a\\ & x\in \left[x^{L},x^{U}\right]\subset R^{n}\\ & s\subset R_{+}^{P-1}\\ & y\in \{0,1{\}}^{u} \end{array}$$

where *f*_1_(**x,y**), *f*_2_(**x,y**), ..., *f*_*P*_(**x,y**) are *P* objective functions to be optimized; *ϵ*_2_, *ϵ*_3_, ..., *ϵ*_*P*_ are the *P* – 1 right-hand-side parameters corresponding to a specific combination of the enforced lower bounds on the objective functions *f*_2_, *f*_3_, ..., *f*_*P*_;**ŝ** is a vector of surplus variables *ŝ*_2_, *ŝ*_3_, ..., *ŝ*_*P*_ used to transform the objective-function *ϵ*-constraints to equality constraints; *r*_2_, *r*_3_, ..., *r*_*P*_ are the ranges of the *P* – 1 objective functions *f*_2_, *f*_3_, ..., *f*_*P*_, i.e., the difference between their largest and smallest possible values; and *κ* is a small number ranging between 10^–3^ and 10^–6^.

### Process Model and Design Constraints

In this formulation, the UNIFAC model^38^ is used to calculate mixture thermodynamics, including the solubility of solid components in the process solvents (solid–liquid equilibrium) and the miscibility of the process solvents (liquid–liquid equilibrium). Several simplifying assumptions are adopted in the process models (e.g., ideal mixing for calculating liquid density, ideal phase behavior for calculating drying energy requirement, and, for the case study explored in this work, temperature-independent heat capacities and heats of vaporization). These choices mainly affect the predicted utility duties and energy-related KPIs and may introduce errors when the system exhibits strong nonideality or when operating conditions span wide ranges of temperature and composition. The quantitative impact of these assumptions is expected to be small because non-idealities are captured in the mass balances. This approach is thus appropriate for early-stage screening. If desired, more detailed models could be used where available to capture temperature and composition effects (e.g., temperature-dependent heat capacity and heat of vaporization correlations).

#### Solvent Assignment and Properties

##### Solvent Assignment Constraints

Assignment constraints are used to formulate the process constraints in terms of solvent identity by relating them to the specific species in the set *S*.

The following constraints ensure that exactly one solvent species from set *S* is assigned to component *s*1 and that at most one solvent species from the same set is assigned to component *s*2:

4 $$\underset{s\in S}{\sum }y_{s1,s}=1$$

5 $$\underset{s\in S}{\sum }y_{s2,s}\leq 1$$

To ensure that a species selected from set *S* is assigned to only one solvent component (i.e., either *s*1 or *s*2), the following constraint is used:

6 $$\underset{q\in Q}{\sum }y_{q,s}\leq 1,\;s\in S$$

##### Solvent Properties

The properties of *s*1 and *s*2 can be derived from the properties of the species in set *S*. The molar masses, densities, boiling points, and melting points of *s*1 and *s*2 are assigned based on the molar mass *MW*_*s*_, density *ρ*_*s*_, experimental boiling point *T*_*b,s*_, and experimental melting point *T*_*m,s*_ of each solvent species *s* in set *S* as follows:

7 $$MW_{q}=\underset{s\in S}{\sum }MW_{s}y_{q,s},\;q\in Q$$

8 $$\rho_{q}=\underset{s\in S}{\sum }\rho_{s}y_{q,s},\;q\in Q$$

9 $$T_{b,q}=\underset{s\in S}{\sum }T_{b,s}y_{q,s},\;q\in Q$$

10 $$T_{m,q}=\underset{s\in S}{\sum }T_{m,s}y_{q,s},\;q\in Q$$

#### Mass, Volume, Mass Fraction, and Mole Fraction

The mass, volume, mass fraction, and mole fraction of component *i* ∈ *C* in stream *t* ∈ *T* are given as follows:

11 $$M_{i,t}=N_{i,t}MW_{i},\;i\in C;t\in T$$

12 $$M_{i,t}=\rho_{t}V_{i,t},\;i\in C;t\in T$$

13 $$w_{i,t}=\frac{M_{i,t}}{\underset{i^{\prime }\in C}{\sum }M_{i^{\prime },t}},\;i\in C;t\in T$$

14 $$x_{i,t}=\frac{N_{i,t}}{\underset{i^{\prime }\in C}{\sum }N_{i^{\prime },t}},\;i\in C;t\in T$$

where *M*_*i,t*_, *N*_*i,t*_, $w_{i,t}$ and *x*_*i,t*_ denote the mass, moles, mass fraction and mole fraction of component *i* ∈ *C* in stream *t* ∈ *T*, respectively, and *MW*_*i*_ represents the molar mass of component *i* ∈ *C*. *ρ*_*t*_ denotes the mixture density of stream *t* ∈ *T,* which is calculated as a function of the pure-component density, *ρ*_*i*_, of each component in the mixture using the following expression, assuming ideal mixture behavior:

15 $$\frac{1}{\rho_{t}}=\underset{i\in C}{\sum }\frac{w_{i,t}}{\rho_{i}}$$

The following constraint is included in the design formulation to ensure that *s*2 is not present in any of the process streams if it is not selected, i.e., if pure solvent *s*1 is selected for synthesis and crystallization:

16 $$x_{s2,t}\leq \underset{s\in S}{\sum }y_{s2,s},\;t\in T$$

#### Unit Models

Material balances must hold across the heat exchanger:

17 $$N_{i,1}=N_{i,2},i\in C$$

The process models proposed by Muhieddine et al.^39^ are used to describe synthesis and crystallization in the end-to-end process, and are presented in the Supporting Information.

The process heat duties used to compute the energy KPI are expressed mathematically by performing energy balances around the process units under the assumption of ideal mixing. These are expressed in Joules per kilogram of API (product **D**).

(1) Heat exchanger duty *Q*_*H*_. The energy balance around the heat exchanger is given by
18 $$Q_{H}=\frac{\underset{i\in C}{\sum }N_{i,1}\int_{T_{1}}^{T_{2}}C_{p_{i}}^{\left(L\right)}dT}{M_{D,15}}$$
  where $C_{p_{i}}^{\left(L\right)}$ is the liquid-phase molar heat capacity of component *i* ∈ *C*.
(2) Reactor duty *Q*_*R*_. The energy balance around the reactor is given by
$$Q_{R}=(\underset{i\in C}{\sum }N_{i,2}\int_{T_{2}}^{T_{R}}C_{p_{i}}^{\left(L\right)}dT+\overset{R}{\underset{r=1}{\sum }}\xi_{r}\Delta H_{R,r}\left(T_{R}\right)+\underset{i\in C}{\sum }N_{i,3}\int_{T_{R}}^{T_{3}}C_{p_{i}}^{\left(L\right)}dT)/M_{D,15}$$ 19
  where *ξ*_*r*_ is the extent of reaction *r* ∈ *R*, *T*_*R*_ is a reference temperature (usually 298.15 K), and Δ*H*_*R,r*_ is the heat of reaction *r* ∈ *R*.
(3) Crystallizer duty $Q_{C}^{-}$ . The energy balance around the crystallizer is given by
$$Q_{C}^{-}=(\underset{i\in C}{\sum }N_{i,5}\int_{T_{5}}^{T_{3}}C_{p_{i}}^{\left(L\right)}dT+N_{D,6}\left(\int_{T_{m,D}}^{T_{3}}C_{p_{D}}^{\left(L\right)}dT+\Delta H_{m,D}\left(T_{m,D}\right)+\int_{T_{5}}^{T_{m,D}}C_{p_{D}}^{\left(S\right)}dT\right))/M_{D,15}$$ 20
  where $C_{p_{D}}^{\left(S\right)}$ , Δ*H*_*m*,**D**_, and *T*_*m*,**D**_ are the solid-phase molar heat capacity, the molar heat of melting, and the melting point of target product **D**, respectively.
(4) Dryer duty *Q*_*D*_. The energy balance around the dryer is given by :
$$Q_{D}=(\underset{i\in V}{\sum }N_{i,14}\left(\int_{T_{12}}^{T_{dew}}C_{p_{i}}^{\left(L\right)}dT+\Delta H_{V_{i}}\right)+\underset{i\in C^{K}}{\sum }N_{i,15}\int_{T_{12}}^{T_{dew}}C_{p_{i}}^{\left(S\right)}dT)/M_{D,15}$$ 21
  In order to calculate the dew point, *T*_*dew*_, of the solvent mixture, the following assumptions are made:
  - The liquid and vapor phases are ideal, i.e., vapor-phase fugacity coefficients and liquid-phase activity coefficients are assumed to be equal to 1;
  -
$\Delta H_{V_{i}}$ is constant;
  - The solid solutes (API and impurities) have very low volatility and fully crystallize upon solvent evaporation, i.e., *N*_*i*,14_ = 0, *i* ∈ *C* ^*K*^.

Accordingly, the following equations are used to estimate *T*_*dew*_:

22 $$K_{i}\left(T\right)=\frac{P_{sat,i}\left(T\right)}{P_{dry}},\;i\in V$$

23 $$\underset{i\in V}{\sum }\frac{x_{i,12}}{K_{i}\left(T_{dew}\right)}=1$$

where *K*_*i*_ is the vapor-liquid equilibrium constant of solvent *i* ∈ *V* and *P*_*sat,i*_ is the saturation pressure of solvent *i* ∈ *V*.

The Clausius–Clapeyron equation is used to estimate *P*_*sat,i*_ (shown here in Pascal):

24 $$\ln P_{sat,i}\left(T\right)=\frac{\Delta H_{V_{i}}}{R_{g}}\left(\frac{1}{T_{b,i}}-\frac{1}{T}\right)+11.5261,\;i\in V$$

In order to lift the assumption of constant $\Delta H_{V_{i}}$ in eq 24, the semi-empirical Antoine equation can be used to estimate *P*_*sat,i*_. However, the Antoine coefficients of some solvents may not cover a sufficiently large temperature range for the application of interest. Furthermore, some solvents may have different sets of Antoine coefficients based on the experimental temperature ranges on which they were regressed. Accordingly, the Clausius–Clapeyron equation, which can be easily embedded in the mathematical formulation of CAM^*b*^D problems, is used, as has been done in previous works.^47,48^

#### Filter Balances

The following material balances apply across the filter:

25 $$N_{i,5}+N_{i,6}-N_{i,7}-N_{i,8}-N_{i,9}=0,\;i\in C$$

26 $$N_{i,9}=0,\;i\in C\backslash \left\{D\right\}$$

27 $$N_{D,6}=N_{D,9}$$

The volume of the filter cake can be calculated as the sum of the volume occupied by the solid and that occupied by the liquid:

28 $$V_{f}^{c}=\frac{M_{D,9}}{\rho_{D}^{S}}+\underset{i\in C}{\sum }V_{i,8}$$

where $\rho_{D}^{S}$ is the density of the solid product **D**.

The volume of solution in the cake constitutes the void volume of the cake. This can be expressed as follows:

29 $$\underset{i\in C}{\sum }V_{i,8}=p_{f}V_{f}^{c}$$

where *p*_*f*_ is a user-defined value of the cake porosity after filtration.

Since the filter acts as a stream-splitter with no phase change, the mole fractions of each component in the liquid streams 5, 7, and 8 are equal:

30 $$x_{i,5}=x_{i,7}=x_{i,8},\;i\in C$$

#### Washing Unit Balances and Equilibrium Constraints

Washing is usually achieved using the crystallization solvent, an antisolvent, or a mixture of both, in several washing stages.^49^ In this work, a single-stage washing unit in which the optimal volume of wash solvent, *V*_*sw*,10_, is identified by the CAM^*b*^D formulation to meet the product purity requirements is considered.

The following material balances apply across the washing unit:

31 $$N_{i,8}+N_{i,9}+N_{i,10}-N_{i,11}-N_{i,12}-N_{i,13}=0,\;i\in C$$

32 $$N_{i,10}=0,\;i\in C\backslash \left\{sw\right\}$$

33 $$N_{i,13}=0,\;i\in C\backslash \left\{D\right\}$$

The volume of the cake after washing can be estimated as the sum of the volumes occupied by the crystalline material and the volumes occupied by solvents *s*1, *s*2, and *sw*:

34 $$V_{w}^{c}=\frac{M_{D,13}}{\rho_{D}^{S}}+\underset{i\in V}{\sum }V_{i,12}$$

Similar to filtration, the volume of solution in the cake after washing constitutes the void volume of the cake. This can be expressed as follows:

35 $$\underset{i\in V}{\sum }V_{i,12}=p_{w}V_{w}^{c}$$

where *p*_*w*_ is a user-defined value of the cake porosity after washing.

The volume of wash solvent used is expressed as a factor of the cake void volume after filtration:

36 $$V_{sw,10}=F_{w}p_{f}V_{f}^{c}$$

where *F*_*w*_ typically ranges between 1 and 5. A factor *F*_*w*_ lower than 1 is not considered in the formulation, as previous studies have shown that more than one cake void volume of wash solvent is typically used to purify the API cake.^49^

Since the washing filter acts as a stream splitter with no phase change, the mole fractions of each component in the liquid streams 11 and 12 are equal:

37 $$x_{i,11}=x_{i,12},\;i\in C$$

Depending on the wash solvent used, the washing step can either partially dissolve the crystallized target product **D** (if the crystallization solvent is used) or cause dissolved impurities to crystallize (if an antisolvent is used). Accordingly, it is important to include equilibrium constraints that ensure that no dissolution of **D** can occur and that all impurities remain undersaturated.

The following constraint enforces that **D** remains at solid–liquid equilibrium in stream 12, i.e., after washing:

38 $$\ln x_{D,12}+\ln\gamma_{D,12}\left(x_{12},T_{12}\right)=\frac{\Delta H_{m,D}}{R_{g}}\left[\frac{1}{T_{m,D}}-\frac{1}{T_{12}}\right]$$

where *x*_**D**,12_ is the mole fraction of product **D** in stream 12; *γ*_**D**,12_ is the liquid-phase activity coefficient of product **D** in stream 12, which is a function of the stream composition **x**_12_ and temperature *T*_12_ and is calculated using UNIFAC; and *R*_*g*_ is the gas constant.

The following constraint enforces that all solid impurities remain under-saturated after washing:

39 $$x_{i,12}\leq {\mathrm{x̂}}_{i,12}^{\left(i\right)}-\epsilon_{s},\;i\in C^{K}\backslash \left\{D\right\}$$

where *ϵ*_*s*_ is a small, positive number and, similar to the crystallization model, ${\mathrm{x̂}}_{12}^{\left(i\right)}$ is a vector of mole fractions in a hypothetical stream 12^*(i)*^ that is similar to stream 12 but saturated in component *i*, *i* ∈ *C* ^*K*^ \ {**D**}. The composition of this stream is obtained by imposing solid–liquid equilibrium for component *i* as follows:

40 $$\ln{\mathrm{x̂}}_{i,12}^{\left(i\right)}+\ln{\mathrm{\gamma ̂}}_{i,12}\left({\mathrm{x̂}}_{12}^{\left(i\right)},T_{12}\right)=\frac{\Delta H_{m,i}}{R_{g}}\left[\frac{1}{T_{m,i}}-\frac{1}{T_{12}}\right],\;i\in C^{K}\backslash \left\{D\right\}$$

where Δ*H*_*m,i*_ is the molar heat of melting of component *i*, *T*_*m,i*_ is the melting point of component *i*, ${\mathrm{\gamma ̂}}_{i,12}\left({\mathrm{x̂}}_{12}^{\left(i\right)},T_{12}\right)$ is the liquid-phase activity coefficient of component *i* in hypothetical stream 12^*(i)*^ and is calculated by UNIFAC, and ${\mathrm{x̂}}_{12}^{\left(i\right)}$ is such that the molar ratios relative to *s*1 of each component except *i* is the same in stream 12 and in the hypothetical stream 12^*(i)*^:

41 $$\frac{{\mathrm{x̂}}_{i^{\prime },12}^{\left(i\right)}}{{\mathrm{x̂}}_{s1,12}^{\left(i\right)}}=\frac{x_{i^{\prime },12}}{x_{s1,12}},i^{\prime }\in C\backslash \left\{s1,i\right\},i\in C^{K}\backslash \left\{D\right\}$$

42 $$\underset{i^{\prime }\in C}{\sum }{\mathrm{x̂}}_{i^{\prime },12}^{\left(i\right)}=1,i\in C^{K}\backslash \left\{D\right\}$$

#### Dryer Balances

The set *V* of process solvents is an extension of the set *Q* that additionally includes the wash solvent, *sw*. In the dryer model, it is assumed that all solvents evaporate and all dissolved solutes, including any impurities that have not been removed by washing, are left behind as solids. Accordingly, the following material balances apply across the

43 $$N_{i,12}+N_{i,13}-N_{i,14}-N_{i,15}=0,i\in C$$

$$N_{i,14}=0,i\in C^{k}$$ 44

45 $$N_{i,15}=0,i\in V$$

Furthermore, the dryer is assumed to operate under atmospheric pressure.

### Process Design Constraints

The proposed CAM^*b*^D formulation includes a set of process-design constraints on the operating temperatures and solvent miscibilities. These include those in the formulation of Muhieddine et al.,^39^ with additional constraints on the miscibility of each of *s*1 and *s*2 with *sw*.

#### Constraints on Operating Temperatures

To ensure that the selected solvents, *s*1 and *s*2, remain in the liquid phase during operation, process stream temperatures are constrained to fall between the boiling and melting points of these solvents. These constraints are formulated via the big-M approach,^44^ using a large constant *M*_*T*_ and a temperature offset *T*_*o*_ to ensure that the process operates sufficiently away from solvent phase-change temperatures to account for possible temperature fluctuations during operation:

46 $$T_{t}-\left(T_{b,q}-T_{o}\right)\leq M_{T}\left(1-\underset{s\in S}{\sum }y_{q,s}\right),q\in Q,t\in T$$

47 $$\left(T_{m,q}+T_{o}\right)-T_{t}\leq M_{T}\left(1-\underset{s\in S}{\sum }y_{q,s}\right),q\in Q,t\in T$$

where the solvent boiling and melting points are calculated using eqs 9 and 10 and *T*_*o*_ is set to 10 K in the current work.

#### Constraints on Solvent Consumption

A minimum solvent consumption of 3.5 kg solvents/kg API in the crystallizer is embedded as a constraint in the CAM^*b*^D formulation.^37,39^ This value corresponds to a slurry with a large solid content, which may pose some operational challenges in the crystallizer. It has been chosen to fully explore the trade-offs between solvent use and other KPIs and can be changed easily if a less concentrated slurry is required (e.g., for continuous crystallization):

48 $$\frac{\underset{i\in Q}{\sum }M_{i,5}}{M_{D,6}}\geq 3.5$$

#### Solvent Miscibility

Solvent miscibility constraints allow for the design of solvent mixtures that form a single liquid phase at the optimal stream compositions and process conditions. Since such constraints cannot be formulated in closed form for mixtures of more than 2 components, an algebraic miscibility constraint for binary mixtures can be used as an approximation. This has been the case, for example, in the works of Buxton et al.,^50^ Karunanithi et al.,^14,15^ and Jonuzaj et al.^18^

For a binary mixture of components *i* and *j*, the following inequality is a sufficient condition for phase stability:

49 $$\frac{\partial^{2}\left(\frac{\Delta G^{mix}}{RT}\right)}{\partial x_{i}^{2}}\geq 0,\forall x_{i}\in \left[0,1\right]$$

where Δ*G* ^mix^ is the molar Gibbs energy change of mixing for the binary system, and *x*_*i*_ is the mole fraction of either component in the mixture. This inequality is equivalent to the following phase stability condition:^51^

50 $$\frac{\partial \ln\gamma_{i}}{\partial x_{i}}+\frac{1}{x_{i}}\geq 0,\forall x_{i}\in \left[0,1\right]$$

where *γ*_*i*_ is the liquid-phase activity coefficient of component *i* in the binary mixture.

Unfortunately, it is very difficult to evaluate this condition because of the need to consider all mole fractions. In practice, it is evaluated at a single point, namely, the composition of interest. If this is not satisfied, then the mixture is unstable. If this is satisfied, the mixture may be stable or metastable. This approach thus eliminates some unsuitable mixtures, but further checks are required to ensure the miscibility of the design obtained.

The mole fraction of solvent *s*1 in a binary mixture of *s*1 and *s*2 with the same *s*1:*s*2 ratio as in stream *t*, is denoted by *x̃*_*s*1,*t*_. It can be calculated using the following expression:

51 $${\mathrm{x̃}}_{s1,t}=\frac{x_{s1,t}}{x_{s1,t}+x_{s2,t}}$$

However, since it is only meaningful to calculate this when a binary solvent mixture is used, this is rewritten as

52 $$-{\mathrm{M̃}}_{x}\left(1-\underset{s\in S}{\sum }y_{s2,s}\right)\leq {\mathrm{x̃}}_{s1,t}-\frac{x_{s1,t}}{x_{s1,t}+x_{s2,t}}\leq {\mathrm{M̃}}_{x}\left(1-\underset{s\in S}{\sum }y_{s2,s}\right),t\in \left\{1,2,3,5\right\}$$

where a big-M formulation is implemented with constant *M̃*_*x*_. The miscibility constraint for the binary pair of solvent components *s*1 and *s*2 is expressed as follows:^51^

53 $$\frac{\partial \ln{\mathrm{\gamma ̃}}_{s1,t}\left({\mathrm{x̃}}_{t},T_{t}\right)}{\partial {\mathrm{x̃}}_{s1,t}}+\frac{1}{{\mathrm{x̃}}_{s1,t}}\geq -M_{\gamma }\left(1-\underset{s\in S}{\sum }y_{s2,s}\right),t\in \left\{1,2,3,5\right\}$$

where $\frac{\partial \ln{\mathrm{\gamma ̃}}_{s1,t}\left({\mathrm{x̃}}_{t},T_{t}\right)}{\partial {\mathrm{x̃}}_{s1,t}}$ is the analytical derivative of the UNIFAC model with respect to *x̃*_*s*1,*t*_, **x̃**_*t*_ is the vector of mole fractions, **x̃**_*t*_ = (*x̃*_*s*1,*t*_, *x̃*_*s*2,*t*_); and *M*_*γ*_ is a sufficiently large constant, which ensures the constraint is only enforced when component *s*2 exists.

Miscibility constraints in stream 12 are also included in the formulation to ensure that each of the identified solvents, *s*1 and *s*2 (if selected), is miscible with *sw*, hence enabling an effective washing procedure.

The mole fraction of solvent *s*1 in a binary mixture of *s*1 and *sw*, *x̃*_*s*1,12_, and the mole fraction of solvent *s*2 in a binary mixture of *s*2 and *sw*, *x̃*_*s*2,12_, are calculated using the following expressions:

54 $${\mathrm{x̃}}_{s1,12}=\frac{x_{s1,12}}{x_{s1,12}+x_{sw,12}}$$

55 $$-{\mathrm{M̃}}_{x}\left(1-\underset{s\in S}{\sum }y_{s2,s}\right)\leq {\mathrm{x̃}}_{s2,12}-\frac{x_{s2,12}}{x_{s2,12}+x_{sw,12}}\leq {\mathrm{M̃}}_{x}\left(1-\underset{s\in S}{\sum }y_{s2,s}\right)$$

where a big-M formulation is implemented for *s*2 with constant *M̃*_*x*_. The miscibility constraints for the binary solvent pairs (*s*1, *sw*) and (*s*2, *sw*) are given by

56 $$\frac{\partial \ln{\mathrm{\gamma ̃}}_{s1,12}\left({\mathrm{x̃}}_{12}^{s1},T_{12}\right)}{\partial {\mathrm{x̃}}_{s1,12}}+\frac{1}{{\mathrm{x̃}}_{s1,12}}\geq 0$$

57 $$\frac{\partial \ln{\mathrm{\gamma ̃}}_{s2,12}\left({\mathrm{x̃}}_{12}^{s2},T_{12}\right)}{\partial {\mathrm{x̃}}_{s2,12}}+\frac{1}{{\mathrm{x̃}}_{s2,12}}\geq -M_{\gamma }\left(1-\underset{s\in S}{\sum }y_{s2,s}\right)$$

where $\frac{\partial \ln{\mathrm{\gamma ̃}}_{q,12}\left({\mathrm{x̃}}_{12}^{q},T_{12}\right)}{\partial {\mathrm{x̃}}_{q,12}}$ is calculated as described above for $\frac{\partial \ln{\mathrm{\gamma ̃}}_{s1,t}\left({\mathrm{x̃}}_{t},T_{t}\right)}{\partial {\mathrm{x̃}}_{s1,t}}$ , ${\mathrm{x̃}}_{12}^{q}$ is a vector of mole fractions *x̃*_*q*,12_ (*q* ∈ *Q*) and *x̃*_*sw*,12_, and *M*_*γ*_ is a sufficiently large constant.

As mentioned above, the miscibility constraints included in the proposed CAM^*b*^D formulation constitute preliminary conditions for a binary solvent mixture to be miscible at the composition and temperature of interest. These constraints, which are are based on eq 49, focus on the apparent miscibility gap (whose range is defined by the points at which the second-order derivative of Δ*G* ^*mix*^/*RT* is zero) and hence do not take the metastable region into account to consider the real miscibility gap.^52^ These constraints also do not consider the impact of non-solvent species (e.g., the API or any byproducts) on the phase stability of the mixture. Accordingly, a rigorous phase stability calculation is required to ensure that any mixture identified as an optimal design is indeed miscible (as a binary or multi-component mixture). Recent work by Karia et al.^53^ and Zhang et al.^54^ has been focused on embedding a surrogate model of the rigorous miscibility test in solvent selection, based on artificial neural networks or support vector machines. This approach has been applied successfully to a three-component system comprising ibuprofen, *s*1 and *s*2.

Occasionally, the identity of solvents *s*1 or *s*2 is found to be the same as that of *sw* at the optimal solution. In such cases, a modified CAM^*b*^D formulation, in which the mole fractions of these solvents are combined when performing equilibrium calculations, must be used. The reader is referred to the Supporting Information for the model equations to be used in this case.

### KPIs

The following KPIs are considered in the end-to-end design formulation:

- Crystal Yield or *Y*_*C*_ (%) is defined as the percentage of **D** crystallized out of solution, expressed in moles:
58 $$Y_{C}=\frac{N_{D,6}}{N_{D,3}}\times 100\%$$
- Process Yield or *Y*_*P*_ (%) is defined as the number of moles of product **D** per moles of limiting reagent A fed to the process, multiplied by a stoichiometric factor SF:
59 $$Y_{P}=\frac{N_{D,15}}{N_{A,1}}\times \mathrm{SF}\times 100\%$$
- Solvent E-factor, or SEF (kg Solvents/kg API), is defined as the mass of solvent consumed throughout the end-to-end process per mass of dried product **D**:
$$\mathrm{SEF}=(\underset{i\in Q}{\sum }M_{i,7}+\underset{i\in Q}{\sum }M_{i,11}+\underset{i\in Q}{\sum }M_{i,14}+M_{sw,11}+M_{sw,14})/M_{D,15}$$ 60
- Process E-factor, or PEF (kg Waste/kg API), is defined as the mass of waste produced in the end-to-end process per mass of dried product **D**:
$$\mathrm{PEF}=(\underset{i\in C}{\sum }M_{i,7}+\underset{i\in C}{\sum }M_{i,11}+\underset{i\in C}{\sum }M_{i,14}+M_{sw,11}+M_{sw,14}+\underset{i\in J}{\sum }M_{i,15})/M_{D,15}$$ 61
- Product Purity or PP (%) is defined as the mass of product **D** per total mass of dried product:
62 $$\mathrm{PP}=\frac{M_{D,15}}{\underset{i\in C^{K}}{\sum }M_{i,15}}\times 100\%$$
- Process energy consumption *Q*_T_ (kJ/kg API) is expressed as the sum of the heat duties of the individual process units. This includes the heat exchanger duty to pre-heat the reactor feed, the reactor duty to maintain a specific reaction temperature, the crystallizer duty to cool the API mixture, and the dryer duty to vaporize the process solvents.
  The energy KPI can be expressed as
63 $$Q_{T}=Q_{H}+\left|Q_{R}\right|+Q_{C}^{-}+Q_{D}$$
  where the absolute value of *Q*_*R*_ and the minus sign in $Q_{C}^{-}$ ensure consistent signs of the energy duties, i.e., *Q*_*H*_, *Q*_*R*_, $Q_{C}^{-}$ and *Q*_*D*_ ≥ 0. This KPI can be minimized in a single or multiobjective optimization problem. The absolute value term in eq 63 can be removed by reformulating the energy KPI objective function as follows:
64 $$\min Q_{H}+Q_{R}^{\prime }+Q_{C}^{-}+Q_{D}$$
  and adding the following two constraints to the full formulation:
65 $$Q_{R}\leq Q_{R}^{\prime }$$
66 $$-Q_{R}\leq Q_{R}^{\prime }$$
  where $Q_{R}^{\prime }$ is a variable used to incorporate the absolute value of *Q*_*R*_ in the expression of *Q*_*T*_.

## Case Study: The Synthesis, Crystallization, and Isolation of Mefenamic Acid

The single-step, end-to-end synthesis of mefenamic acid (MA), a non-steroidal anti-inflammatory drug with analgesic properties, from 2,3-dimethylaniline (DMA) and 2-chlorobenzoic acid (CBA) is a simple process that can be used as a case study to illustrate the solvent selection approach. The overall reaction scheme is shown in Figure 2, excluding any inorganic or ionic species, e.g., HCl, involved in the synthesis.

**Figure 2:**
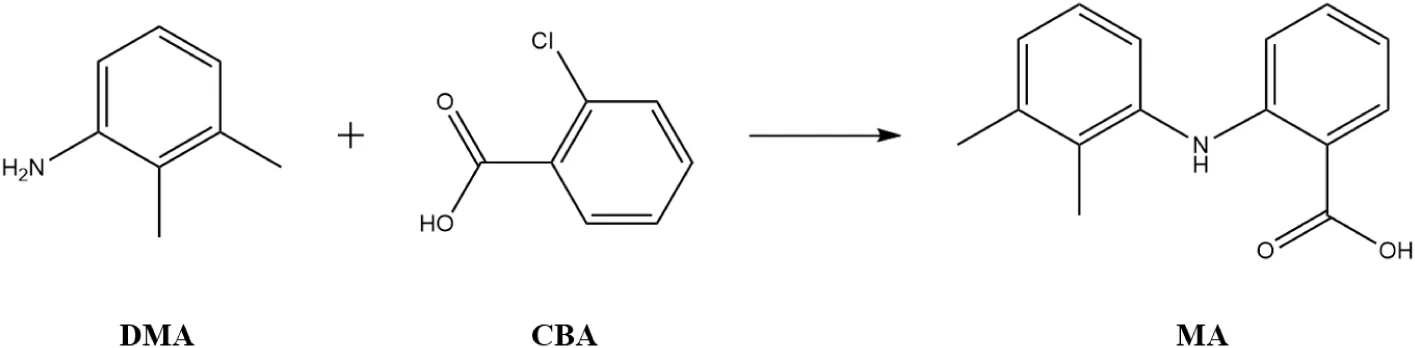
Reaction of 2,3-dimethylaniline (DMA) and 2-chlorobenzoic acid (CBA) to form mefenamic acid (MA).

To apply the proposed general model to this case study, the following sets are specified: *C* = {DMA, CBA, MA, *s*1, *s*2}, *C* ^K^ = {DMA, CBA, MA}, and *Q* = {*s*1, *s*2}. CBA and MA are thus treated as impurities that may impact several of the KPIs. The CAM^*b*^D formulation identifies solvent mixtures of at most 2 components that optimize the selected KPIs. Seven different CAM^*b*^D problems are formulated. The first four consist of single-objective problems: in the first formulation, the SEF is minimized; in the second, third, and fourth formulations, *Y*_P_ is maximized while considering different design formulations (restricted and flexible). The remaining three formulations are multiobjective optimizations: in the fifth formulation, the SEF is minimized while PP is maximized; in the sixth formulation, *Q*_T_ is minimized while *Y*_P_ is maximized and in the seventh formulation, the SEF, *Y*_P_, and PP are simultaneously optimized (min SEF, max *Y*_P_, and max PP).

All formulations include the set of process model equations describing the material and energy balances across the process units (eqs 17, (S.1, S.2, S.7–S.9), ([Disp-formula eq18]–[Disp-formula eq24]), ([Disp-formula eq25]–[Disp-formula eq30], 31–37, ([Disp-formula eq43]–[Disp-formula eq45]), as well as equilibrium constraints describing species solubility, i.e., under-saturation of CBA during synthesis, crystallization, and washing (eqs 39–42) (DMA exists in the liquid phase at the process operating temperatures); under-saturation of MA during synthesis (Equations S.3–S.6) and that MA is at solid–liquid equilibrium during crystallization (Equation S.10) and washing (eq 38) (Equations S.3–S.6) (Equations S.11–S.14). The formulations also include a set of design specifications, such as process temperature constraints to ensure solvents are in the liquid phase during operation (eqs 46 and 47), miscibility constraints to reduce the likelihood of a second liquid phase appearing (eqs 51–57) and constraints on the target values/ranges of the selected KPIs, such as the solvent consumption constraint in eq 48. In this case study, *n*-heptane is used as the wash solvent due to the limited solubility of MA in this solvent.^37^ The main model equations and design specifications are given in Table 1, whereas the model parameters are provided in Table 2. A production target of 80 kg of mefenamic acid is set arbitrarily. Since all KPIs are expressed on a per kg of API basis and capital costs (unit sizes) are not taken into account, this does not affect the optimal solutions. It can be seen that the final crystallization temperature range has been narrowed down from the range used in Muhieddine et al.^39^ for the integrated synthesis and crystallization case from 290 K ≤ *T*_5_ ≤ 380 K to 290 K ≤ *T*_5_ ≤ 306 K so that *T*_5_ is close to the washing temperature *T*_12_ = 298.15 K, hence reducing the possibility of undesired solute precipitation. Solvents *s*1 and *s*2 are selected from a set of solvents commonly used in the pharmaceutical industry.^37,39^ These solvents are listed in the Supporting Information. Depending on the design formulation and the availability of data on solvent thermodynamic properties, one of two lists, List I (42 candidates) or List II (49 candidates), as provided in the Supporting Information, is considered. In the first five design problems, the optimal solvents are selected from List I. In the sixth and seventh design problems, the optimal solvents are selected from List II. This represents a large number of discrete designs. For List I, there are 42 possible single-solvent processes (with cooling crystallization), 861 possible processes with two solvents (the solvent order does not matter), and 3,444 processes with two solvents and antisolvent crystallization with or without cooling (where *s*2 is the antisolvent). This yields a total of 4,347 design options (5,929 for List II), not taking into account the choice of operating conditions.

**Table 1:** Main CAM*^b^* D Model Equations and Design Specifications of the Mefenamic Acid Case Study

Constraint	Equation	Allowed KPI Range (where applicable)	Units (where required)
Solvent E-factor	$\mathrm{SEF}=\frac{\underset{i\in Q}{\sum }M_{i,5}+M_{Heptane,10}}{M_{MA,15}}$	3.5–20	kg Solvents/kg API
Process E-factor	$\mathrm{PEF}=\frac{\underset{i\in C}{\sum }M_{i,5}+M_{Heptane,10}}{M_{MA,15}}$	3.5–20	kg Waste/kg API
Crystal Yield	$Y_{C}=\frac{N_{MA,6}}{N_{MA,3}}\times 100\%$	90–100	%
Process Yield	$Y_{P}=\frac{N_{MA,15}}{N_{CBA,1}}\times 100\%$	75–95	%
Product Purity	$PP=\frac{M_{MA,15}}{\underset{i\in C^{K}}{\sum }M_{i,15}}\times 100\%$	95–100	%
Feed temperature	*T*_1_ = 298.15	–	K
Isothermal reactor operation	*T*_2_ = *T*_3_	–	K
2:1 DMA:CBA Feed Ratio	*N*_*DMA*,2_ = 2*N*_*CBA*,2_	–	mol
No MA in process feed	*N*_*MA,1*_ = 0	–	mol
Reaction conversion	*x* ^*c*^ = 90	–	%
Throughput	*M*_*MA*,15_ = 80	–	kg
Reaction temperature range	358 ≤ *T*_2_ ≤ 403	–	K
Final crystallization temperature range	290 ≤ *T*_5_ ≤ 306	–	K
Dryer pressure	*P*_*dry*_ = 1	–	bar
Cake porosity (Filtration)	*p*_*f*_ = 0.4	–	–
Cake porosity (Washing)	*p*_*w*_ = 0.4	–	–

**Table 2:** Parameters for the CAM*^b^* D Formulation of the Mefenamic Acid Case Study

Parameter	Value	Units (where required)
*T*_*o*_	10	K
*M*_*T*_	600	–
*M̃*_*x*_	10	–
*M*_*γ*_	100	–
*SF*	1	mol/mol

In all energy balances, heat capacity and heat of vaporization are assumed to be weak functions of temperature, i.e., standard constant-pressure heat capacities (of the chemical compounds) and constant heats of vaporization (of the solvent candidates) are used. Standard constant-pressure heat capacities and heats of vaporization of solvent candidates in set *S* are obtained from the NIST Chemistry WebBook (https://webbook.nist.gov/chemistry), whereas standard constant-pressure heat capacities of reactants and products are calculated using the GC method of Kolská et al.^55^ Furthermore, heat capacity is assumed to be phase independent, i.e., $C_{p_{i}}^{\left(L\right)}\approx C_{p_{i}}^{\left(S\right)}$ for *i* ∈ *C* ^*K*^. In addition, pure-component solid densities *ρ*_*i*_ for *i* ∈ *C* ^*K*^ are obtained from the Chemical Book (https://www.chemicalbook.com/ProductIndex_EN.aspx). All property values used in the formulations are reported in the Supporting Information.

The optimization problems are solved in GAMS version 32.2.0 ^56^ using the local MINLP solver SBB.^57^ Accordingly, the reported solutions should be viewed as locally optimal for the selected initial guess and problem formulation, rather than as guaranteed global optima. Because the MINLP formulation is nonconvex, different starting points can converge to different local minima. To mitigate this, the workflow can be run from multiple initializations (multistart), with solution quality evaluated by reporting the best objective values found across runs. In this study, the emphasis is on assessing the feasibility of an optimization-based approach to solvent selection and process design for end-to-end drug substance manufacturing, while acknowledging that identifying globally optimal solutions for systems of this complexity remains challenging. Integer cuts are included in the MINLP formulation to generate multiple optimal solutions for each design problem that are ranked with respect to the objective function values. For each solution of a single-objective design problem, the values of SEF, *Y*_P_, and PP are reported. For each solution of a bi-objective design problem, the values of the objective functions are reported. In addition, for each solution of all single- and bi-objective design problems, the following are reported: the identities of solvent components *s*1 and *s*2, the reaction temperature *T*_2_, the final crystallization temperature *T*_5_, the composition of the solvent in the reactor, defined as the ratio of the mole fraction of solvent 1 to that of solvent 2 in the reactor outlet (stream 2), and the composition of the solvent in the crystallizer, defined as the ratio of the mole fraction of solvent 1 to that of solvent 2 in the crystallizer outlet (stream 5). For each solution of the three-objective design problem, the solvent identities and values of the objective functions are reported. For each problem, ten solutions are reported, unless less than ten feasible solutions are identified by the solver, in which case all feasible solutions are given.

### Minimizing the SEF

The first CAM^*b*^D formulation involves minimizing the SEF. Ten optimal solutions, ranked in order of increasing SEF, are listed in Table 3. Anisole appears recurrently as a favorable solvent. Alcohols (butanol/propanol), nitrobenzene, and acetates also appear in several solutions. This list provides a set of promising candidates that must be evaluated further. For instance, acetic acid may react with DMA, leading to a loss of yield and formation of new impurities. The extent and impact of this issue would, therefore, need to be investigated. It can be seen that solutions 1, 6, 7, 8, 9, and 10 correspond to hybrid cooling and antisolvent crystallization, as is evident in the temperature decrease from *T*_2_ to *T*_5_ and the change in the ratio of solvent mole fractions across crystallization, whereas all other solutions correspond to cooling crystallization. Furthermore, most solutions achieve approximately the same SEF, with values ranging between 3.81 and 3.83 kg/kg. This narrow range of SEF values does not offer a clear distinction among the reported solutions. Accordingly, it would be useful to consider alternative objective functions, such as the process yield, *Y*_P_, to generate a ranked list of distinct solutions from which better insights on optimal solvent selection and process design can be derived.

**Table 3:** Ten Optimal Solutions of the CAM*^b^* D Problem with the Objective of Minimizing the Solvent E-Factor (SEF)

Solution	Solvents	SEF (kg/kg API)	*Y*_*P*_ (%)	PP (%)	*T*_2_ (K)	*T*_5_ (K)	$\frac{x_{s1,2}}{x_{s2,2}}$	$\frac{x_{s1,5}}{x_{s2,5}}$
1	s1: n-Butanol	3.81	81.95	95.13	381	306	0.25	0.24
	s2: Nitrobenzene							
2	s1: 2-Pentanol	3.82	81.93	95.22	380	306	0.32	0.32
	s2: Nitrobenzene							
3	s1: Anisole	3.82	81.83	95.76	376	306	2.36	2.36
	s2: n-Butanol							
4	s1: Nitrobenzene	3.82	83.53	95.31	382	306	1.01	1.01
	s2: Anisole							
5	s1: Anisole	3.82	82.52	95.56	374	306	1.08	1.08
	s2: Acetic acid							
6	s1: 2-Pentanol	3.82	82.58	95.81	380	306	0.45	0.44
	s2: Anisole							
7	s1: Cyclohexanone	3.83	84.73	95.70	403	306	0.11	0.06
	s2 : Anisole							
8	s1: n-Butyl acetate	3.83	84.90	95.74	389	306	0.11	0.09
	s2: Anisole							
9	s1: Isobutyl acetate	3.83	84.90	95.74	389	306	0.11	0.09
	s2: Anisole							
10	s1: Cyclohexanone	3.83	85.58	95.10	403	306	0.11	0.07
	s2: Nitrobenzene							

Several of the solvent pairs identified are similar to those reported in Muhieddine et al.^39^ for the problem of minimizing the SEF in the integrated synthesis and crystallization of MA; these include anisole/n-butanol, anisole/acetic acid, cyclohexanone/anisole, isobutyl acetate/anisole, cyclohexanone/nitrobenzene, and *n*-butyl acetate/anisole. However, while the aforementioned solutions achieve the best possible objective function value of 3.5 kg solvent/kg API in the integrated synthesis and crystallization process, their performance varies in terms of optimizing the process-wide SEF, as reported in Table 3. This shows that designs that are found to be optimal for a subset of unit operations, e.g., synthesis and crystallization, do not necessarily perform as well when assessed from a process-wide perspective, i.e., by considering synthesis, crystallization, and isolation and including KPIs that account for this extension in the CAM^*b*^D formulation.

The inclusion of interlinked design decisions in the CAM^*b*^D formulation allows the identification of solvents that not only form a miscible mixture but are also miscible with the wash solvent, *n*-heptane. Had the solvent selection problem been solved sequentially, for example, by identifying optimal solvent identities for an integrated synthesis and crystallization process and then fixing those identities before optimizing the end-to-end process, solvents that form an immiscible mixture with *n*-heptane, such as pure water, could have been identified, leading to ineffective washing with limited impurity rejection.

### Maximizing *Y_P_*

Crystallization can have a significant influence on process performance, given the interrelationship between crystallization performance—i.e., crystal yield—and process-wide KPIs such as throughput, process yield, and energy consumption. In order to explore the effect of crystallization on process-wide performance in terms of process yield, the second, third, and fourth design formulations involve maximizing *Y*_P_ while considering different crystallization conditions (fixed or variable operating temperatures, with or without antisolvent addition) and comparing the results obtained for each of them.

#### Restricted Design Problem 1: Fixed Crystallization Temperatures with Antisolvent Addition Allowed

The second CAM^*b*^D formulation involves maximizing *Y*_P_ while fixing the initial crystallization (or reaction) temperature *T*_2_ to its upper bound, i.e., 403 K, and the final crystallization temperature *T*_5_ to its lower bound, i.e., 290 K, and allowing antisolvent addition to occur. This reflects the common practice of using as large of temperature drop as possible in the crystallizer as a means of maximizing yield. Therefore, this problem includes restrictions on the process operating temperatures and on the flexibility in the crystallizer mode of operation (only cooling or hybrid cooling/antisolvent crystallization are possible).

The solver was not able to generate more than five solutions for this design problem. This shows that fixing decision variables in the CAM^*b*^D formulation, instead of solving an integrated design problem, can have the undesirable effect of reducing the number of feasible solutions. The top five solutions, generated in order of decreasing *Y*_*P*_, are listed in Table 4. Solution 1 corresponds to a cooling crystallization process, whereas solutions 2–5 involve cooling and antisolvent addition. The downside of fixing the operating temperatures is that it restricts the design space of process solvents to those whose liquid range falls within 403 K and 290 K. Accordingly, potentially promising solvents that could result in equal or better objective function values than those obtained by solving this restricted formulation, as well as solvents that may exhibit high SHE performance, such as n-butyl acetate,^9^ are no longer feasible. Strikingly, no solutions containing alcohols appear in the top 5 candidates, indicating the strong interaction between the KPI chosen as the objective and the corresponding solutions. A high overall yield is achieved in all cases, but the SEF, which is no longer considered during optimization, deteriorates significantly.

**Table 4:** Five Optimal Solutions of the CAM*^b^* D Problem with the Objective of Maximizing Process Yield (*Y_P_*) with Fixed Crystallization Temperatures and Antisolvent Addition Allowed

Solution	Solvents	*Y*_*P*_ (%)	SEF (kg/kg API)	PP (%)	*T*_2_ (K)	*T*_5_ (K)	$\frac{x_{s1,2}}{x_{s2,2}}$	$\frac{x_{s1,5}}{x_{s2,5}}$
1	s1: Nitrobenzene	88.12	3.85	95.82	403	290	0.24	0.24
	s2: Cyclohexanone							
2	s1: Anisole	87.93	3.85	95.97	403	290	0.11	0.07
	s2: Cyclohexanone							
3	s1: Anisole	87.64	5.28	98.33	403	290	0.11	0.07
	s2: Nitrobenzene							
4	s1: 1,2-Propanediol	87.40	5.28	98.61	403	290	0.11	0.06
	s2: Isobutyl acetate							
5	s1: 1,2-Propanediol	86.73	5.28	98.55	403	290	0.11	0.05
	s2: Anisole							

#### Restricted Design Problem 2: Variable Crystallization Temperatures with No Antisolvent Addition Allowed

The third CAM^*b*^D formulation involves maximizing *Y*_*P*_ while allowing *T*_2_ and *T*_5_ to vary in a purely cooling crystallization process, i.e., no antisolvent addition is allowed. This condition can be enforced in the mathematical formulation by the inclusion of *N*_*s*2,4_ = 0 as a design constraint. Therefore, this problem includes restrictions on the crystallizer mode of operation (only cooling crystallization allowed) but flexibility in setting the process operating temperatures. Ten optimal solutions, ranked with respect to *Y*_*P*_, are listed in Table 5. It can be seen that solvent components with superior SHE performance, such as *n*-butyl acetate, can now be identified due to the removal of the operating temperature constraint. Four out of the ten solutions result in *Y*_*P*_ ≥ 88%, none of which has an initial crystallization temperature at the upper bound of 403 K. In comparison, only one of the solutions identified by solving Restricted Design Problem 1 results in *Y*_*P*_ ≥ 88%; additionally, looking at the nitrobenzene/cyclohexanone design (solution 1) in Tables 4 and 5, it can be seen that both a *T*_2_ of 403 and 391 K result in the same *Y*_*P*_, SEF, and PP. This can be explained by the fact that the crystallization yield limit could have already been reached due to the properties of the solvents and the solutes. Accordingly, there is no point in using the maximum temperature drop for this particular design, as this increases process costs without improving any of the KPIs. This emphasizes the importance of implementing an integrated and flexible approach to solvent selection and process design. Solving Restricted Design Problem 2 hence offers solutions that are superior to those obtained by solving Restricted Design Problem 1.

**Table 5:** Ten Optimal Solutions of the CAM*^b^* D Problem with the Objective of Maximizing Process Yield (*Y_P_*) with Variable Crystallization Temperatures and No Antisolvent Addition Allowed

Solution	Solvents	*Y*_*P*_ (%)	SEF (kg/kg API)	PP (%)	*T*_2_ (K)	*T*_5_ (K)	$\frac{x_{s1,2}}{x_{s2,2}}$	$\frac{x_{s1,5}}{x_{s2,5}}$
1	s1: Nitrobenzene	88.12	3.85	95.82	391	290	0.24	0.24
	s2: Cyclohexanone							
2	s1: Nitrobenzene	88.10	5.58	98.56	389	290	2.10	2.10
	s2: *n*-Butyl acetate							
3	s1: Nitrobenzene	88.10	5.56	98.56	389	290	2.15	2.15
	s2: Isobutyl acetate							
4	s1: *n*-Butyl acetate	88.00	7.15	97.86	389	290	9.00	9.00
	s2: Isobutyl acetate							
5	s1: Anisole	87.91	5.28	98.62	392	290	0.11	0.11
	s2: Cyclohexanone							
6	s1: Anisole	87.85	7.07	98.95	389	290	0.11	0.11
	s2: *n*-Butyl acetate							
7	s1: Anisole	87.83	5.28	98.63	389	290	1.18	1.18
	s2: Isobutyl acetate							
8	s1: Cyclohexanone	87.80	7.38	99.03	389	290	0.11	0.11
	s2: *n*-Butyl acetate							
9	s1: Isobutyl acetate	87.80	7.34	99.03	362	290	9.00	9.00
	s2: Cyclohexanone							
10	s1: t-AmOH	86.28	11.36	98.71	365	290	0.90	0.90
	s2: *n*-Butanol							

#### Flexible Design Problem: Variable Crystallization Temperatures with Antisolvent Addition Allowed

The fourth CAM^*b*^D formulation involves maximizing *Y*_P_ while allowing *T*_2_ and *T*_5_ to vary and considering the possibility of antisolvent addition. Ten optimal solutions, ranked with respect to *Y*_*P*_, are listed in Table 6. Solutions 5 and 10 correspond to a hybrid cooling and antisolvent crystallization process, as shown by both the decrease in temperature from *T*_2_ to *T*_5_ and the decrease in the ratio of solvent mole fractions between synthesis and crystallization. All other solutions correspond to cooling crystallization, as shown by the drop in temperature and the constant ratio of solvent mole fractions across the crystallizer. Since most solutions, including the top four, are cooling crystallization designs, it can be said that antisolvent use for this case study is not as crucial/impactful as cooling to induce product crystallization and meet the design objectives. It can be seen that in all solutions, the SEF is greater than the lower bound of 3.5 kg/kg and the difference between *T*_2_ and *T*_5_ is wide: *T*_2_ is close to its upper bound of 403 K in most solutions, and *T*_5_ is at its lower bound of 290 K in all ten solutions. This can be explained by the demanding process requirements that favor process yield maximization. Several of the reported solutions are identical to those reported in Table 5, indicating that both the Restricted Design Problem 2 and the Flexible Design Problem formulations yield similar results, and that the impact of antisolvent use on the selected KPI target values for this case study is small. Specifically, designs including nitrobenzene/*n*-butyl acetate, nitrobenzene/isobutyl acetate, anisole/*n*-butyl acetate, and anisole/isobutyl acetate are identical in terms of solvent choices and process conditions in the two formulations, whereas designs including nitrobenzene/cyclohexanone, *n*-butyl acetate/isobutyl acetate, anisole/cyclohexanone, and cyclohexanone/*n*-butyl acetate share the same solvent identities but different process conditions (temperatures + mole fractions), which impact the selected KPIs; for example, in solution 1 in Table 5 (nitrobenzene/cyclohexanone), the design corresponds to an SEF of 3.85 kg/kg with minimal wash solvent use (*F*_*w*_ = 1) and a PP of 95.82%, whereas in solution 1 in Table 6, the design corresponds to an SEF of 5.29 kg/kg and a PP of 98.56% due to greater wash solvent addition (*F*_*w*_ = 5). However, each of these two designs could have been identified by any of the two formulations since they both correspond to cooling crystallization. An interesting finding is that the product purity constraint is not active at the reported solutions in Table 6, and a PP greater than 95% can be achieved when generating different solutions. Thus, a product purity of over 95% does not appear to be a demanding target in this process.

**Table 6:** Ten Optimal Solutions of the CAM*^b^* D Problem with the Objective of Maximizing Process Yield (*Y_P_*) with Variable Crystallization Temperatures and Antisolvent Addition Allowed

Solution	Solvents	*Y*_*P*_ (%)	SEF (kg/kg API)	PP (%)	*T*_2_ (K)	*T*_5_ (K)	$\frac{x_{s1,2}}{x_{s2,2}}$	$\frac{x_{s1,5}}{x_{s2,5}}$
1	s1: Nitrobenzene	88.15	5.29	98.56	403	290	0.26	0.26
	s2: Cyclohexanone							
2	s1: Nitrobenzene	88.10	5.58	98.56	389	290	2.10	2.10
	s2: n-Butyl acetate							
3	s1: Nitrobenzene	88.10	5.56	98.56	389	290	2.15	2.15
	s2: Isobutyl acetate							
4	s1: n-Butyl acetate	88.00	7.96	98.98	389	290	9.00	9.00
	s2: Isobutyl acetate							
5	s1: Anisole	87.96	5.28	98.62	403	290	0.11	0.07
	s2: Cyclohexanone							
6	s1: Anisole	87.85	7.07	98.95	389	290	0.11	0.11
	s2: n-Butyl acetate							
7	s1: Anisole	87.83	5.28	98.63	389	290	1.18	1.18
	s2: Isobutyl acetate							
8	s1: Cyclohexanone	87.78	6.27	97.56	389	290	0.11	0.11
	s2: n-Butyl acetate							
9	s1: n-Butanol	87.62	7.57	99.22	381	290	0.11	0.11
	s2: 2-Pentanol							
10	s1: Acetic acid	83.94	9.24	99.30	362	290	6.50	4.13
	s2: 2,2,4-Trimethylpentane							

It can be seen that those KPIs that are not an objective function may vary significantly from one solution to another, and it is therefore interesting to explore the competing relationships or trade-offs between those KPIs through multiobjective optimization. This is considered in subsequent sections, where hybrid cooling and antisolvent crystallization (the flexible design problem) are considered in all cases.

### Multiobjective Optimization: Exploring Trade-Offs between Competing KPIs

#### Minimizing the SEF and Maximizing PP

To better explore the trade-offs between the KPIs, the fifth CAM^*b*^D formulation is a bi-objective optimization (BOO) problem that involves minimizing the SEF and maximizing PP. The problem is solved using the *ϵ*-constraint method,^45^ in which the SEF is minimized while PP is constrained by a given lower bound *ϵ* ranging between 98.6% and 99.4%. The Pareto front representing the Pareto-optimal solutions of this problem is shown in Figure 3, and the details of each solution are given in Table 7. It can be seen that a marginal increase in purity beyond 98.8% requires a significant increase in the SEF. Since the Pareto curve before PP = 98.8% is relatively flat, indicating a small increase in solvent consumption with increasing purity, the solution corresponding to (SEF,PP) = (5.45,98.8) would be a good compromise solution. It can also be seen that some solutions correspond to the same solvent identities and process temperatures, such as anisole/n-butanol in solutions 2 and 3 and isobutyl acetate/n-butanol in solutions 5 and 6, yet to different solvent proportions in the process units; this clearly influences the SEF, PP, and *Y*_*P*_ and highlights the capability of CAM^*b*^D to generate different designs based on the KPI of interest. Furthermore, the types of solvent mixtures generated through BOO are noticeably different from those obtained in single-objective problems, with much greater prevalence of primary alcohols, which appear in every solution. The consideration of two KPIs places greater demands on the properties of the solvents, pushing the optimizer toward different parts of the design space.

**Table 7:** Pareto-Optimal Solutions of the Bi-Objective Optimization (BOO) Problem for Minimizing the Solvent E-Factor (SEF) and Maximizing Product Purity (PP)

Solution	*ϵ* (%)	Solvents	SEF (kg/kg API)	PP (%)	*Y*_*P*_ (%)	*T*_2_ (K)	*T*_5_ (K)	$\frac{x_{s1,2}}{x_{s2,2}}$	$\frac{x_{s1,5}}{x_{s2,5}}$
1	98.60	s1: Nitrobenzene	5.17	98.60	86.84	382	290	0.28	0.28
		s2: 2-Pentanol							
2	98.70	s1: Anisole	5.21	98.70	87.11	381	290	0.16	0.16
		s2: *n*-Butanol							
3	98.80	s1: Anisole	5.45	98.80	87.21	381	290	0.13	0.13
		s2: *n*-Butanol							
4	98.90	s1: Cyclohexanone	5.80	98.90	87.23	381	290	0.12	0.12
		s2: *n*-Butanol							
5	99.00	s1: Isobutyl acetate	6.22	99.00	87.17	381	290	0.11	0.10
		s2: *n*-Butanol							
6	99.10	s1: Isobutyl acetate	6.79	99.10	86.98	381	290	0.11	0.09
		s2: *n*-Butanol							
7	99.20	s1: Cyclohexanone	7.54	99.20	86.60	381	290	0.11	0.08
		s2: *n*-Butanol							
8	99.30	s1: *n*-Butanol	8.33	99.30	87.42	381	290	0.11	0.10
		s2: 2-Pentanol							
9	99.40	s1: Isobutanol	9.52	99.40	86.57	371	290	4.26	4.26
		s2: *n*-Butanol							

**Figure 3:**
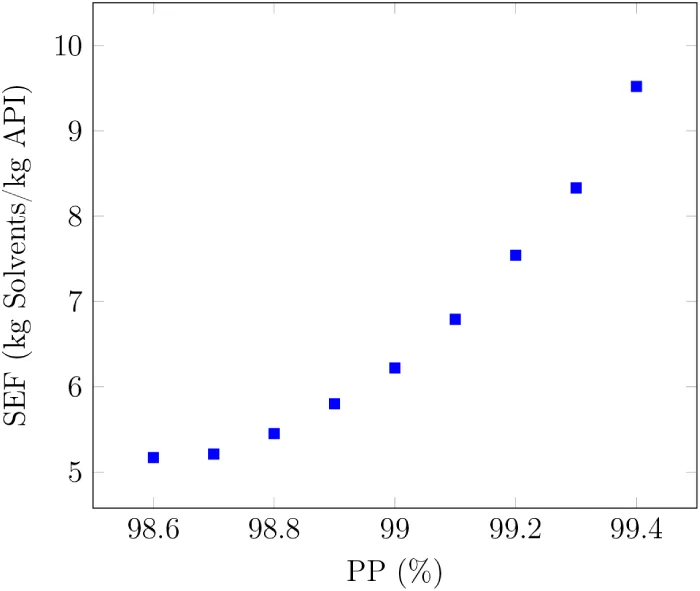
Pareto front of the bi-objective optimization (BOO) problem for minimizing the Solvent E-factor (SEF) (kg solvent/kg API) and maximizing Product Purity (PP) (%).

#### Minimizing *Q_T_* and Maximizing *Y_P_*

It is expected that higher process yields, which are linked to higher crystal yields, require higher process energy consumption; for example, significant temperature drop requirements during crystallization (higher *Q*_*H*_ and $Q_{C}^{-}$ ) may contribute significantly to *Q*_*T*_. Accordingly, the sixth formulation of the end-to-end design problem involves minimizing *Q*_*T*_ and maximizing *Y*_*P*_ to explore the competing relationship between these two KPIs. This BOO problem is solved using the *ϵ*-constraint method, in which *Q*_*T*_ is minimized while *Y*_*P*_ is constrained by a given lower bound *ϵ,* which is varied between 81% and 89%. The Pareto front representing the Pareto-optimal solutions of this problem is shown in Figure 4, and the details of each solution are given in Table 8. An interesting finding is that all solutions, except solution 4, involve hybrid cooling and antisolvent crystallization. As in the previous formulation, a further change in the types of solvent mixtures selected is also observed, with all 7 species selected appearing as solutions for the first time. It can be seen that an increase in *Y*_*P*_ from 85% to 87% requires a significant increase in *Q*_*T*_ from 1502 kJ/kg API to 1967 kJ/kg API; accordingly, the design point (*Q*_*T*_,*Y*_*P*_) = (1502,85) would be a good compromise solution in this case. Overall, this formulation highlights the critical importance of the choice of design criteria and the exploration of interactions between solvent, process, and multiple criteria.

**Table 8:** Pareto-Optimal Solutions of the Bi-Objective Optimization (BOO) Problem for Minimizing Process Energy Consumption (*Q_T_*) and Maximizing Process Yield (*Y_P_*)

Solution	*ϵ* (%)	Solvents	*Q*_*T*_ (kJ/kg API)	*Y*_*P*_ (%)	*Y*_*C*_ (%)	*T*_2_ (K)	*T*_5_ (K)	$\frac{x_{s1,2}}{x_{s2,2}}$	$\frac{x_{s1,5}}{x_{s2,5}}$
1	81	s1: 1,4-Dioxane	1315	82	90.00	364	301	1.70	1.14
		s2: Formic acid							
2	83	s1: DMAC	1342	83	91.58	364	297	9.00	0.53
		s2: o-Xylene							
3	85	s1: DMAC	1502	85	93.80	384	292	1.10	0.36
		s2: Chlorobenzene							
4	87	s1: Toluene	1967	87	96.45	374	290	3.23	3.23
		s2: DMF							
5	89	s1: Chlorobenzene	2922	89	98.86	394	297	9.00	1.40
		s2: o-Xylene							

**Figure 4:**
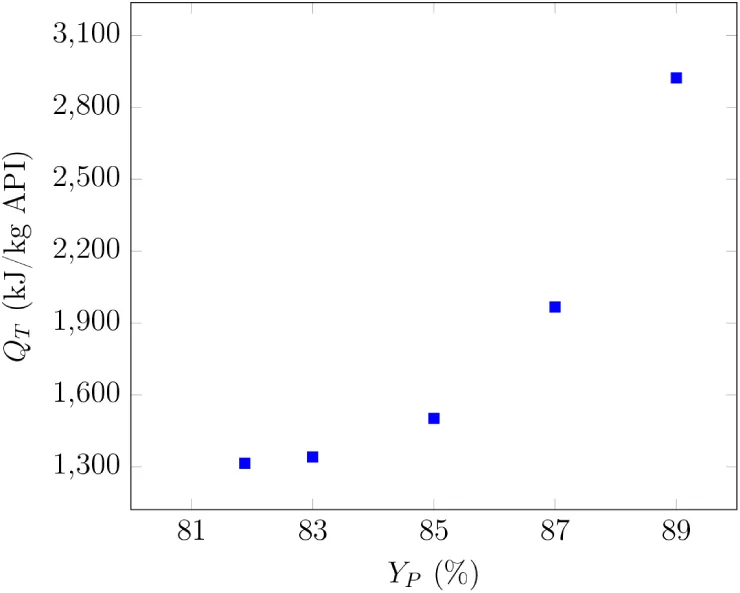
Pareto front of the bi-objective optimization (BOO) problem for minimizing Process Energy Consumption (*Q_T_*) (kJ/kg API) and maximizing Process Yield (*Y_P_*) (%).

Figure 5 shows the energy-related contributions of the process units to *Q*_*T*_, where *Q*_*H*_ and *Q*_*R*_ are combined in a single contribution (heating the reactor feed + maintaining isothermal reactor operation), with values reported in Table 9. It can be seen that $Q_{C}^{-}$ is the largest of all energy contributions in each of the reported solutions, and increases with increasing *Y*_*P*_; hence, $Q_{C}^{-}$ is the main reason for the competing relationship between *Q*_*T*_ and *Y*_*P*_. This is expected since a greater *Y*_*P*_ generally requires a greater *Y*_*C*_ (see Table 8) as more product needs to be crystallized from solution and sent to the isolation steps. This is driven by larger temperature drops in the crystallizer and/or antisolvent addition, leading to higher $Q_{C}^{-}$ requirements. *Q*_*H*_, while of smaller magnitude, also increases rapidly with *Y*_*C*_. Finally, it can be seen that the *Q*_*D*_ values of the reported solutions are close to each other and are significantly less than their $Q_{C}^{-}$ counterparts; this is because there is a significant reduction in the amount of reaction/crystallization solvent(s) that reaches the dryer due to the two filtration stages involved in the end-to-end process. Accordingly, the *Q*_*D*_ values mainly correspond to the energy required to vaporize the wash solvent, *n*-heptane, which is the main component of the solvent mixture entering the dryer (stream 12), as seen in Table 10.

**Table 9:** Process Unit Energy Contributions and the Total Process Energy Consumption for the Five Reported Pareto-Optimal Solutions in Table 8

Solution	*Q*_*H*_(kJ/kg API) + |*Q*_*R*_| (kJ/kg API)	*Q*_*C*_^−^ (kJ/kg API)	*Q*_*D*_(kJ/kg API)	*Q*_*T*_ (kJ/kg API)
1	166	785	364	1315
2	160	818	364	1342
3	110	951	441	1502
4	448	1102	417	1967
5	715	1810	397	2922

**Figure 5:**
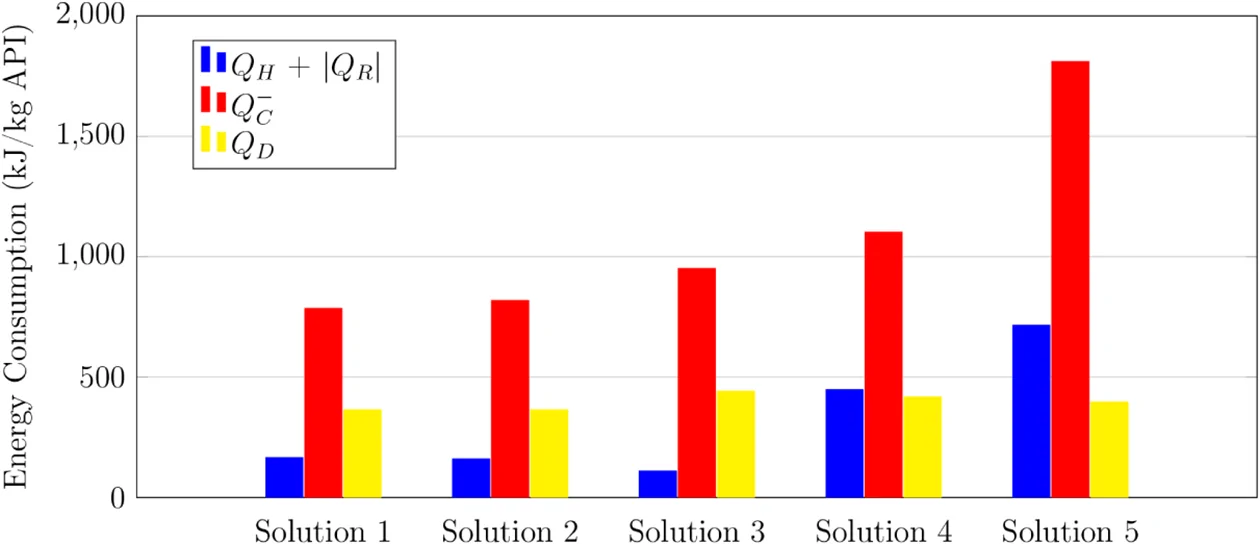
Process unit energy contributions to the total Process Energy Consumption KPI (*Q_T_*) per kg of API for the five reported Pareto-optimal solutions in Table 8.

**Table 10:** Number of Moles of Reaction/Crystallization Solvents s1, s2, and Wash Solvent *sw* in Stream 12, and Wash Solvent Fraction of Cake Void Volume *F_w_* Values of the Reported Pareto-Optimal Solutions of the Bi-Objective Optimization (BOO) Problem Involving Process Energy Consumption (*Q_T_*) and Process Yield (*Y_P_*)

Solution	Solvent *q* ∈ *V*	*N*_*q*,12_ (moles in absolute amounts)	*F*_*w*_ (dimensionless)
1	s1: 1,4-Dioxane	47.03	5.00
	s2: Formic acid	41.20	
	*sw*: *n*-Heptane	239.50	
2	s1: DMAC	21.56	4.22
	s2: o-Xylene	40.57	
	*sw*: *n*-Heptane	232.43	
3	s1: DMAC	22.80	3.00
	s2: Chlorobenzene	62.61	
	*sw*: *n*-Heptane	215.93	
4	s1: Toluene	81.17	2.48
	s2: DMF	25.13	
	*sw*: *n*-Heptane	204.65	
5	s1: Chlorobenzene	34.70	5.00
	s2: o-Xylene	24.69	
	*sw*: *n*-Heptane	239.17	

In order to better understand solvent effects on process energy consumption, the molar flows of solvents *s*1, *s*2, and *sw*, which are the main components of the process streams, in the end-to-end process of solution 4 and that of solution 5 (Table 8) are shown in Figure 6. The number of moles (in absolute amounts) highlighted in blue, red, and yellow correspond to those being subject to heating or cooling in the heat exchanger/reactor, crystallizer, and dryer, respectively. The larger solvent quantities entering the heat exchanger/reactor in solution 5 relative to solution 4 contribute to the larger reactor duty in solution 5. Similarly, the larger solvent quantities being subject to cooling in the crystallizer in solution 5, which involves antisolvent addition (*N*_*s*2,4_ ≠ 0), relative to solution 4 (cooling crystallization), contribute to the larger crystallizer duty in solution 5. However, the larger solvent quantities entering the dryer in solution 4 relative to solution 5 contribute to the slightly larger dryer duty in solution 4, even though washing in solution 5 involves a larger amount of wash solvent (*F*_*w*_ = 5.00) relative to solution 4 (*F*_*w*_ = 2.48). It should also be noted that, in addition to solvent molar quantities, solvent properties such as heat capacity and heat of vaporization contribute to the process unit duties, as clearly seen in the corresponding previously derived expressions of *Q*_*H*_ (eq 18), *Q*_*R*_ (eq 19), $Q_{C}^{-}$ (eq 20), and *Q*_*D*_ (eq 21).

**Figure 6:**
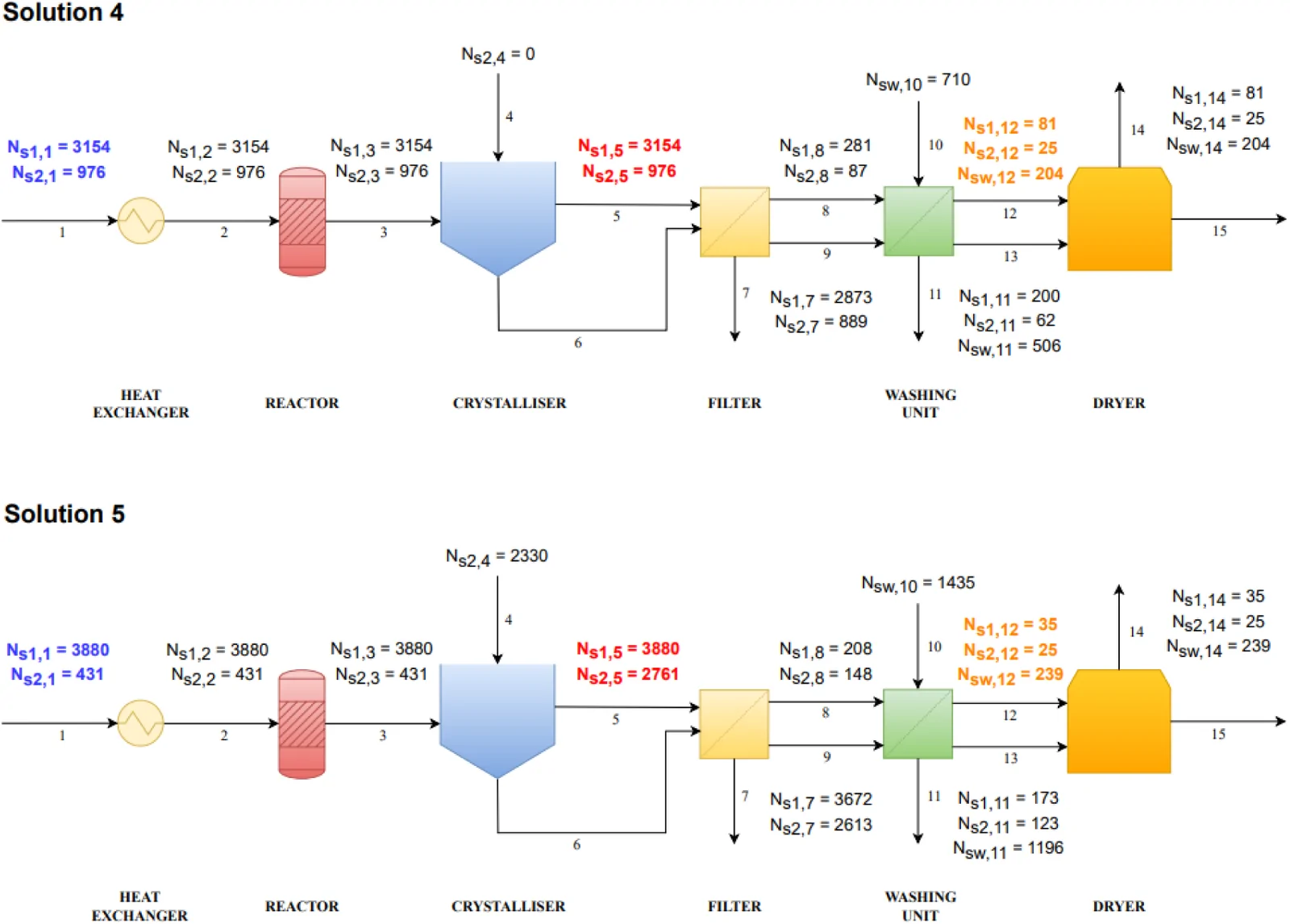
The number of moles, in absolute amounts and rounded to the nearest integer, of reaction/crystallization solvents *s*1, *s*2 and wash solvent *sw* in the end-to-end processes corresponding to solutions 4 and 5 in Table 8. Quantities highlighted in blue, red, and yellow are those contributing to the energy duty of the heat exchanger/reactor, crystallizer, and dryer, respectively.

#### Minimizing the SEF, Maximizing *Y*_P_, and Maximizing PP

All of the presented MOO design problems so far were limited to 2 objective functions. In order to explore the applicability of the proposed methodology to more than 2 objectives, the seventh and final formulation of the end-to-end design problem involves minimizing the SEF, maximizing *Y*_P_, and maximizing PP. This MOO problem is solved using AUGMENCON, where the SEF is set as the main objective function while *Y*_*P*_ and PP are transformed into constraints, as demonstrated in the Problem Definition and Formulation section. The corresponding mathematical formulation of the objective functions can hence be represented as follows:

67 $$\begin{array}{ll} \max & -SEF+\kappa \left(\frac{s_{2}}{r_{2}}+\frac{s_{3}}{r_{3}}\right)\\ \mathrm{s.t.} & Y_{P}-s_{2}=\epsilon_{2}\\ & \mathrm{PP}-s_{3}=\epsilon_{3} \end{array}$$

where *κ* is set to 10^–3^ and the ranges *r*_2_ and *r*_3_ are determined by recording the values of the objective functions at the optimal solution of each of the three single-objective optimization problems. These values are provided in Table 11 where it can be seen that 81.80% ≤ *ϵ*_2_ ≤ 88.10% and 95.70% ≤ *ϵ*_3_ ≤ 99.60%. Five values for each of *ϵ*_2_ and *ϵ*_3_ are considered by dividing their respective ranges into four equal intervals. Accordingly, this MOO problem involves solving 25 instances of the problem to cover the different combinations of *ϵ*_2_ and *ϵ*_3_.

**Table 11:** Optimal KPI Values Used in Determining the Objective Function Ranges in the Multiobjective Optimization (MOO) Problem Involving the Solvent E-Factor (SEF), Process Yield (*Y_P_*), and Product Purity (PP)

	KPI		
Objective function	SEF (kg/kg API)	*Y*_*P*_ (%)	PP (%)
Min SEF	3.82	81.80	95.70
Max *Y*_*P*_	5.29	88.10	98.60
Max PP	12.82	86.00	99.60

Plots of the Pareto-optimal solutions of this MOO problem are displayed in Figure 7 and are labeled by solution number. In each of the plots, the Pareto-optimal solutions are represented by the values of two KPIs and are color-coded by the value of the third KPI. The solutions are detailed in table form in the Supporting Information.

**Figure 7:**
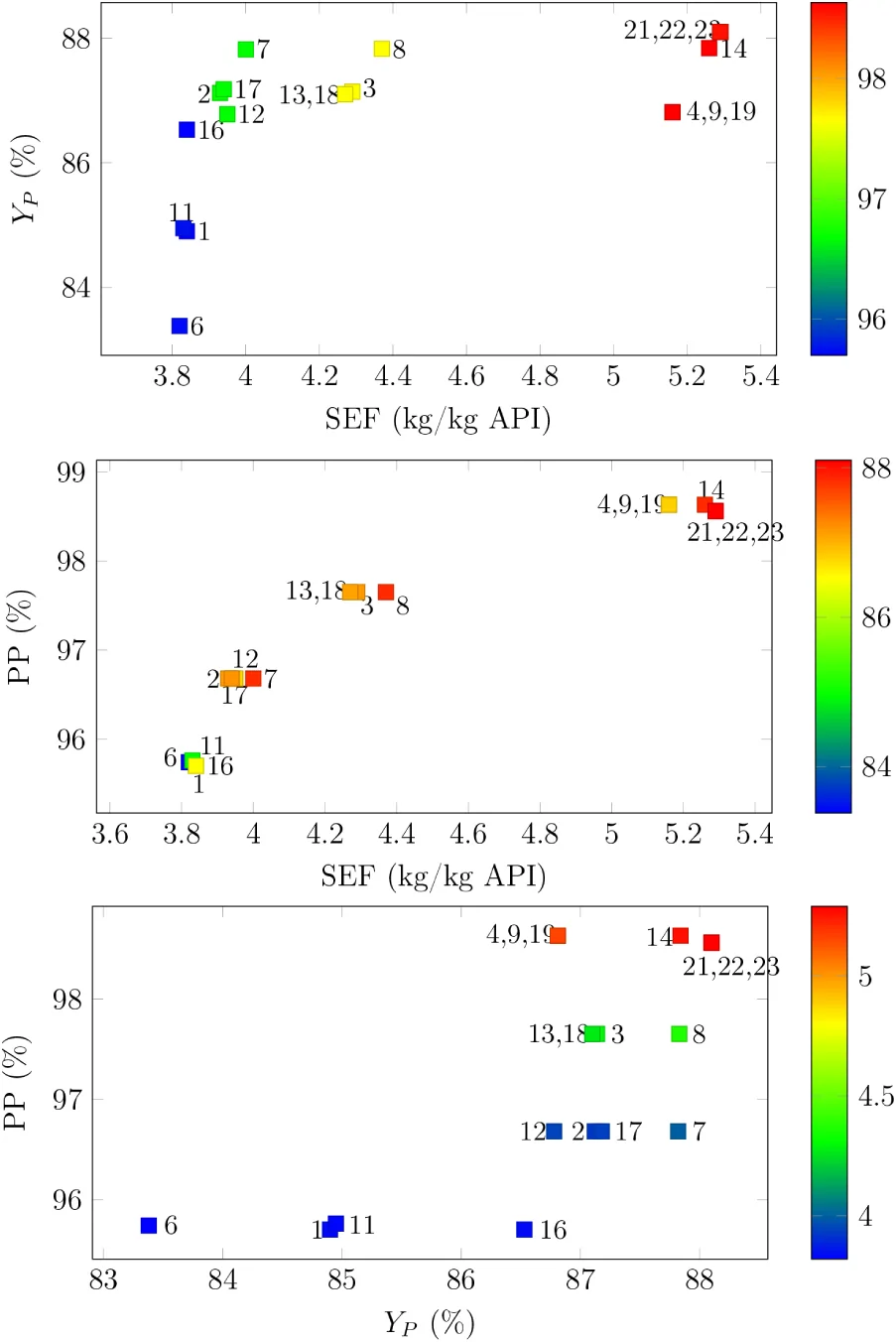
Plots of the Pareto-optimal solutions of the multiobjective optimization (MOO) problem involving the Solvent E-factor (SEF), Process Yield (*Y_P_*) and Product Purity (PP). The solutions are labeled by solution number as given in the Supporting Information. The color scale in each plot corresponds to the KPI value not represented on either axes of that plot.

It can be seen that there is an inverse relationship between the SEF and PP. This is expected, as achieving higher purity requires the consumption of additional amounts of wash solvent. The relationship between *Y*_*P*_ and the other two KPIs is not as straightforward. In general, the relationship between *Y*_*P*_ and PP is direct, since a larger amount of solid-phase API throughout the process would contribute to a high *Y*_*P*_ and, if that amount is significantly larger than that of impurities, to a high PP. However, at very high purity, the large use of wash solvent (large SEF) may lead to product loss if more API is rejected with the impurities. This could result in a reduction in *Y*_*P*_ if the ratio of the solid-phase API molar quantity to that of the limiting reagent decreases, even if PP is increased due to a further reduction in the amount of impurities relative to that of the API. This explains why the 2-pentanol/anisole design (solutions 4, 9, and 19) exhibits a slight decrease in *Y*_*P*_ relative to the preceding solution. Ultimately, the relationship between the SEF, *Y*_P_, and PP strongly depends on the chemical and process system considered; for example, a solvent mixture that induces high crystal yield of the API may lead to enhanced *Y*_*P*_ even at high PP levels. This is the case in solution 14 (anisole/isobutyl acetate with *Y*_*C*_ = 97.36%), which exhibits an improvement in PP and *Y*_*P*_ but a deterioration in SEF relative to the previous solutions for which *ϵ*_2_ = 84.95%. Some *ϵ*_2_-*ϵ*_3_ combinations lead to infeasible solutions due to high PP targets (solutions 5, 10, 15, and 20), as well as high *Y*_*P*_ and PP targets (solutions 24 and 25). For *ϵ*_2_ = 88.10% and *ϵ*_3_ = 95.70%, 96.68%, and 97.65% (solutions 21, 22, and 23), the same solution, i.e., nitrobenzene/cyclohexanone, is identified. It is, in fact, the solution reported in Table 11 for the Max *Y*_*P*_ problem.

## Conclusion

A systematic solvent selection approach for the integrated synthesis, crystallization, and isolation of APIs was presented. The proposed CAM^*b*^D formulation makes it possible to identify a ranked list of solvent mixtures, mixture compositions, and process conditions that optimize selected KPIs while meeting comprehensive design specifications such as the miscibility of the reaction/crystallization solvent(s) with the wash solvent, and solubility constraints across synthesis, crystallization, filtration, and washing. The approach is based on a versatile mixed-integer nonlinear multiobjective framework that facilitates the consideration of a large number of design options, the investigation of a range of KPIs, and the introduction of new models and design considerations. The solution of the MINLP provides a diverse list of solvent and process designs that can be further investigated, potentially accelerating process development.

The approach was illustrated by identifying optimal solvent and process designs for the end-to-end production of mefenamic acid. Furthermore, three multiobjective CAM^*b*^D design problems were formulated to explore the trade-offs between competing KPIs, namely the solvent E-factor (SEF) and product purity (PP); process energy consumption (*Q*_*T*_) and process yield (*Y*_*P*_); and SEF, *Y*_P_, and PP. Some interesting findings were observed; for example, solving the SEF/PP bi-objective design problem generated different designs with varying SEF/PP values, showing that a PP of 98.8% can be achieved without significantly deteriorating the SEF. Additionally, solving the *Q*_*T*_/*Y*_*P*_ bi-objective design problem showed that the crystallizer energy consumption, $Q_{C}^{-}$ , was the greatest contributor to the total energy consumption, *Q*_*T*_, of the end-to-end process, due to the link between high crystal yield and process yield maximization. Finally, solving the SEF/*Y*_*P*_/PP multiobjective design problem highlighted some interesting trade-offs that exist between the three objective functions, particularly those between *Y*_*P*_ and the other two KPIs.

More generally, our work has highlighted that the list of promising solvents depends strongly on the KPIs considered, highlighting the importance of taking a holistic view of solvent selection that includes multiple process units and criteria. This opens many avenues for future work. First, the formulation can be expanded to include inorganic and ionic species, e.g., by using the SAFT-*γ* Mie equation of state for electrolytes;^58,59^ including operational considerations, such as the impact of solid content in the slurry on crystallizer performance or limiting the steepness of the solubility curve with respect to temperature to avoid problems with seeding, or importantly, by making the choice of wash solvent an additional decision variable. An important consideration is the availability of predictive models that relate solvent identity to process-relevant properties (thermodynamics, kinetics). The lack of such models can limit the applicability of the approach. There is thus a need to develop such models further so that the approach can be applied to more APIs and solvents and so that more detailed models can be embedded within the proposed framework. Moreover, we will introduce uncertainty quantification and stochastic optimization in future work in order to provide more insights into the most promising solvent combinations.

Additionally, solvent selection in the present framework is driven by process performance metrics, and we have not explicitly penalized toxicity or broader sustainability considerations. As a result, some of the ranked solutions include solvents that are typically considered nongreen solvents (e.g., nitrobenzene), and these options should be interpreted as process-feasible candidates rather than given an endorsement from a safety, health, and environmental (SHE) perspective. In future work, we will therefore expand the approach to include SHE metrics, as has been done in previous work^37,39^ via the GSK solvent guide,^9,10^ as well as life cycle assessment. This would give insights into the trade-offs between process performance and sustainability and would help to prioritize more sustainable alternatives when multiple solvents offer similar process performance.

Future work will also focus on using CAM^*b*^D-based approaches to evaluate synthetic routes in a given reaction network, based on process performance metrics, including process economics and resource consumption. Current approaches to synthetic route selection are mostly based on experimental findings, with little to no consideration of process performance metrics. This can lead to the identification of routes that perform optimally on the lab scale but poorly at the process or production level. Accordingly, a model-based approach to process route selection would be an invaluable tool to support design decisions in early-stage process development.

## Supplementary Material



## Notes

Process model equations for the reaction and crystallization steps; the UNIFAC thermodynamic model equations; lists of candidate solvents considered in this work (Lists I and II); physical properties of solid species involved in MA synthesis; the standard heat of reaction of MA synthesis; physical properties of solvent candidates considered in this work; Pareto-optimal solutions of the MOO problem involving the SEF, *Y*_P_, and PP; and model equations related to solubility and miscibility that can be used in the CAM^*b*^D formulation in case *s*1 or *s*2 are identical to the wash solvent *sw* (PDF)

## Sets

C: Set of components that may appear in any of the liquid streams in the process
*C* ^*K*^: Set of components that may appear in solid form in the process
J: Set of components that may appear in solid form as impurities in the final dried product
K: Set of UNIFAC functional groups
Q: Set of reaction/crystallization solvents *s*1 and *s*2
R: Set of reaction indices
S: Set of candidate compounds from which solvents *s*1 and *s*2 are selected
V: Set of process solvents *s*1, *s*2 and *sw*
T: Set of process streams

## Symbols

Δ*G* ^mix^: Molar Gibbs energy change of mixing of a binary mixture (J/mol)
Δ*H*_*m,i*_: Molar heat of melting of component *i* ∈ C^*K*^ in process stream *t* ∈ *T* (J/mol)
Δ*H*_*R,r*_: Molar heat of reaction *r* ∈ *R* (J/mol)
$\Delta H_{V_{i}}$: Molar heat of vaporization of solvent species *i* ∈ *V* (J/mol)
$\gamma_{i,t}^{C},\gamma_{i,t}^{R}$: Combinatorial and residual parts of the activity coefficient of component *i* ∈ *C* in process stream *t* ∈ *T*
*γ*_*i,t*_: Liquid-phase activity coefficient of component *i* ∈ C^*K*^ in process stream *t* ∈ *T*
*γ̂*_*i,t*_: Liquid-phase activity coefficient of component *i* ∈ C^*K*^ in a stream that is similar to stream *t* ∈ *T* but saturated in *i*
${\mathrm{x̂}}_{i,t}^{\left(i\right)}$: Mole fraction of component *i* ∈ *C* ^*K*^ in a stream that is similar to stream *t* ∈ *T* but saturated in *i*
*ν*_*i,r*_: Stoichiometric coefficient of component *i* ∈ *C* in reaction *r* ∈ *R*
*ρ*_*i*_: Density of component *i* ∈ *C* (g/mL)
*ρ*_*s*_: Density of solvent species *s* ∈ *S* (g/mL)
*ρ*_*t*_: Density of process stream *t* ∈ *T* (g/mL)
*M̃*_*x*_, *M*_*γ*_: Big M parameters in the solvent miscibility constraints
*x̃*_*s*1,12_: Mole fraction of *s*1 in a binary mixture of *s*1 and *sw* in stream 12
*x̃*_*s*1,*t*_: Mole fraction of *s*1 in a binary mixture of *s*1 and *s*2 in stream *t* ∈ {1,2,3,5}
*x̃*_*s*2,12_: Mole fraction of *s*2 in a binary mixture of *s*2 and *sw* in stream 12
*ξ*_*r*_: Extent of reaction *r* ∈ *R*
$C_{p_{i}}^{\left(L\right)}$: Liquid-phase molar heat capacity of component *i* ∈ *C* (J/mol-K)
$C_{p_{API}}^{\left(S\right)}$: Solid-phase molar heat capacity of the API (J/mol-K)
*F*_*w*_: Factor of cake void volume (1–5 cake void volumes)
*K*_*i*_: Vapor-liquid equilibrium value of solvent component *i* ∈ *V*
*M*_*i,t*_: Mass of component *i* ∈ *C* in process stream *t* ∈ *T* (g)
*M*_*T*_: Big M parameter in the constraints on operating temperatures
*MW*_*i*_: Molar mass of component *i* ∈ *C* (g/mol)
*MW*_*s*_: Molar mass of solvent species *s* ∈ *S* (g/mol)
*n*_*i,k*_: Number of occurrences of functional group *k* ∈ *K* in component *i* ∈ *C*
*N*_*i,t*_: Moles of component *i* ∈ *C* in process stream *t* ∈ *T* (mol)
*n*_*s,k*_: Number of occurrences of functional group *k* ∈ *K* in solvent species *s* ∈ *S*
*P*_*dry*_: Operating pressure of the dryer (bar)
*p*_*f*_: User-defined value of the cake porosity after filtration
*P*_*sat,i*_: Saturation pressure of solvent component *i* ∈ *V* (Pa)
*p*_*w*_: User-defined value of the cake porosity after washing
$Q_{R}^{\prime }$: Variable used to incorporate the absolute value of *Q*_*R*_ in the expression of *Q*_*T*_ (J)
$Q_{C}^{-}$: Crystallizer duty (J)
*Q*_*D*_: Dryer duty (J)
*Q*_*H*_: Heat exchanger duty (J)
*Q*_*R*_: Reactor duty (J)
*Q*_*T*_: Process energy consumption (kJ/kg API)
*q*_*i*_: van der Waals surface area for component *i* ∈ *C*
*Q*_*k*_: Group surface area parameter of functional group *k* ∈ *K*
*R*_*g*_: Universal gas constant, 8.314 (J/mol-K)
*r*_*i*_: van der Waals volume for component *i* ∈ *C*
*R*_*k*_: Group volume parameter of functional group *k* ∈ *K*
*T*_0_: Reference temperature used in the dryer energy balance (K)
*T*_*b,k*_: Contribution of functional group *k* ∈ *K* to the selected solvent boiling point T_*b,q*_ (K)
*T*_*b,q*_: Boiling point of solvent component *q* ∈ *Q* (K)
*T*_*b,s*_: Boiling point of solvent species *s* ∈ *S* (K)
*T*_*dew*_: Dew point of the solvent mixture being evaporated (K)
*T*_*m,i*_: Melting point of component *i* ∈ C^*K*^ in process stream *t* ∈ *T* (K)
*T*_*m,k*_: Contribution of functional group *k* ∈ *K* to the selected solvent melting point T_*m,q*_ (K)
*T*_*m,q*_: Melting point of solvent component *q* ∈ *Q* (K)
*T*_*m,s*_: Melting point of solvent species *s* ∈ *S* (K)
*T*_*o*_: Temperature offset used in the constraints on operating temperatures (K)
*T*_*R*_: Reference temperature used in the reactor energy balance (= 298.15 K)
*T*_*t*_: Temperature of process stream *t* ∈ *T* (K)
$V_{f}^{c}$: Volume of cake after filtration (mL)
$V_{w}^{c}$: Volume of cake after washing (mL)
*V*_*i,t*_: Volume of component *i* ∈ *C* in process stream *t* ∈ *T* (mL)
*w*_*i,t*_: Mass fraction of component *i* ∈ *C* in process stream *t* ∈ *T*
*x* ^*c*^: Reaction conversion
$x_{i,t}^{eq}$: Solid-liquid equilibrium mole fraction of component *i* ∈ C^*K*^ in process stream *t* ∈ *T*
*x*_*i,t*_: Mole fraction of component *i* ∈ *C* in process stream *t* ∈ *T*
*Y*_*C*_: Crystal Yield (%) *Y_P_* Process Yield (%)
*y*_*q,s*_: Binary variable representing the assignment of solvent species *s* ∈ *S* to solvent component *q* ∈ *Q*
CBA: 2-chlorobenzoic acid
DMA: 2,3-dimethylaniline
MA: Mefenamic acid
PEF: Process E-factor (kg Waste/kg Crystals)
PP: Product Purity (%)
SEF: Solvent E-factor (kg Solvent/kg Crystals)
SF: Stoichiometric factor (mol/mol)
SHE: Safety, Health, and the Environment
