# Supplementary material for: Computer-Aided Solvent Selection for the Design of Integrated Synthesis, Crystallization, and Isolation Processes
Source: Org Process Res Dev. 2026 Sep 24;30(10):2672–93. doi: 10.1021/acs.oprd.6c00168 (PMC13693823; doi:10.1021/acs.oprd.6c00168)
Supplement: Supplementary file 1 [file op6c00168_si_001.pdf]

Supporting Information

Computer-aided solvent selection for the design of integrated synthesis, crystallisation and isolation processes

Mohamad H. Muhieddine,† Suela Jonuzaj,†,⊥ Oliver Watson,† Sara Ottoboni,‡,¶ Jan Sefcik,‡,¶ Chris J. Price,‡,¶ Shekhar K. Viswanath,§ Alan Armstrong,∥ Amparo Galindo,† George Jackson,† and Claire S. Adjiman*,†

†*Department of Chemical Engineering, The Sargent Centre for Process Systems Engineering, Imperial College London, South Kensington Campus, London SW7 2AZ, United Kingdom*

‡*Department of Chemical and Process Engineering, University of Strathclyde, Glasgow G1 1XQ, United Kingdom*

¶*EPSRC Centre for Innovative Manufacturing in Continuous Manufacturing and Crystallisation, University of Strathclyde, Glasgow G1 1RD, United Kingdom*

§*Lilly Research Laboratories, Indianapolis, IN 46082, USA*

∥*Department of Chemistry and Institute for Molecular Science and Engineering, Imperial College London, Molecular Sciences Research Hub, White City Campus, Wood Lane, London W12 0BZ, United Kingdom*

⊥*Current address: Lilly Research Laboratories, Indianapolis, IN 46082, USA*

E-mail: c.adjiman@imperial.ac.uk

Reactor and Crystalliser Models

Reactor balances and equilibrium constraints

The synthesis model proposed by Muhieddine et al.[1] is used to describe synthesis in the end-to-end process, and is presented here for completeness. The material balances across the reactor are given by:

$$N_{i,3} = N_{i,2} + \sum_{r \in R} \nu_{i,r} \xi_r, \quad i \in C, \tag{S.1}$$

where $\nu_{i,r}$ is the stoichiometric coefficient of component $i \in C$ in reaction $r \in R$ and ξ_r is the extent of reaction $r \in R$. In the simplest case of a single reaction, with limiting reactant A, the extent of reaction ξ_1 is given by:

$$\xi_1 = x^c N_{\mathrm{A},2}, \tag{S.2}$$

where x^c is the reaction conversion.

The identified process solvents must be able to effectively dissolve all species in set C^K, i.e., all reactants, impurities and products, and maintain a homogeneous liquid stream throughout the reaction. The following constraint is used to enforce species under-saturation:

$$x_{i,t} \le \hat{x}_{i,t}^{(i)} - \epsilon_s, \quad i \in C^K; t = \{2, 3\}, \tag{S.3}$$

where $\hat{x}_{i,t}^{(i)}$ is the solubility of component i in a hypothetical stream that is similar to stream t but saturated in i, and ϵ_s is a small, positive number.

Considering the simple case where a liquid phase is in equilibrium with one crystalline phase only, the solubility of a component $i \in C^K$ in stream t can be computed as follows:[2,3]

$$\ln \hat{x}_{i,t}^{(i)} + \ln \hat{\gamma}_{i,t}(\hat{\mathbf{x}}_t^{(i)}, T_t) = \frac{\Delta H_{m,i}}{R_g} \left[\frac{1}{T_{m,i}} - \frac{1}{T_t} \right], \quad i \in C^K, \quad t \in \{2, 3\}, \tag{S.4}$$

where $\hat{\gamma}_{i,t}$ is the liquid-phase activity coefficient of component i in the hypothetical stream

corresponding to stream t, $\Delta H_{m,i}$ is the molar heat of melting of component i, T_m is the melting point of component i, T_t is the temperature of stream t, R_g is the gas constant, and for each $i \in C^K$, $\hat{\mathbf{x}}_t^{(i)}$ is a mole fraction vector satisfying the following relations:

$$\frac{\hat{x}_{i',t}^{(i)}}{\hat{x}_{s1,t}^{(i)}} = \frac{x_{i',t}}{x_{s1,t}}, \;\; i' \in C \setminus \{s1, i\}, \;\; i \in C^K, \;\; t \in \{2, 3\}, \tag{S.5}$$

$$\sum_{i' \in C} \hat{x}_{i',t}^{(i)} = 1, \;\; i \in C^K, \;\; t \in \{2, 3\}. \tag{S.6}$$

The above equations indicate that the relative amounts of all components but i are the same between stream t and its hypothetical stream, while component i is at solid-liquid equilibrium. The relative amount of component i is calculated to correspond to its solid-liquid equilibrium mole fraction.

The UNIFAC model[2,4] is used to estimate the liquid-phase activity coefficients of components in set C^K. The relevant UNIFAC equations are listed in Appendix .

Crystalliser Balances and Equilibrium Constraints

The crystallisation model proposed by Muhieddine et al.[1] is used to describe crystallisation in the end-to-end process, and is also presented here for completeness. The model neglects the impact of crystallisation kinetics and assumes that the crystalliser operates at thermodynamic equilibrium.

The following material balances apply across the crystalliser, assuming an impurity rejection of 100%:

$$N_{i,3} + N_{i,4} - N_{i,5} - N_{i,6} = 0, \;\; i \in C, \tag{S.7}$$

$$N_{i,4} = 0, \;\; i \in C \setminus \{s2\}, \tag{S.8}$$

$$N_{i,6} = 0, \;\; i \in C \setminus \{\mathbf{D}\}. \tag{S.9}$$

The solubility of target product $\mathbf{D}$ in liquid stream 5 is computed as follows:

$$\ln x_{\mathbf{D},5} + \ln \gamma_{\mathbf{D},5}(\mathbf{x}_5, T_5) = \frac{\Delta H_{m,\mathbf{D}}}{R_g}\left[\frac{1}{T_{m,\mathbf{D}}} - \frac{1}{T_5}\right], \tag{S.10}$$

where $\gamma_{\mathbf{D},5}(\mathbf{x}_5, T_5)$ is estimated using UNIFAC.

To ensure that only $\mathbf{D}$ crystallises while all other solutes in set C^K remain under-saturated, i.e., meeting the 100% impurity rejection requirement, the following constraint is used:

$$x_{i,5} \leq \hat{x}_{i,5}^{(i)} - \epsilon_s, \quad i \in C^K \setminus \{\mathbf{D}\}, \tag{S.11}$$

where ϵ_s is a small, positive number and $\hat{\mathbf{x}}_5^{(i)}$ is a vector of mole fractions in a hypothetical stream that is similar to stream 5 but saturated in component i, $i \in C^K \setminus \{\mathbf{D}\}$, whose solid-liquid equilibrium mole fraction is computed as follows:

$$\ln \hat{x}_{i,5}^{(i)} + \ln \hat{\gamma}_{i,5}(\hat{\mathbf{x}}_5^{(i)}, T_5) = \frac{\Delta H_{m,i}}{R_g}\left[\frac{1}{T_{m,i}} - \frac{1}{T_5}\right], \quad i \in C^K \setminus \{\mathbf{D}\}, \tag{S.12}$$

where $\hat{\gamma}_{i,5}(\hat{\mathbf{x}}_t^{(i)}, T_5)$ is estimated by UNIFAC and $\hat{\mathbf{x}}_5^{(i)}$ is such that the relative proportions of all components in set C^K but $\mathbf{D}$ are the same between stream 5 and the hypothetical stream:

$$\frac{\hat{x}_{i',5}^{(i)}}{\hat{x}_{s1,5}^{(i)}} = \frac{x_{i',5}}{x_{s1,5}}, \quad i' \in C \setminus \{s1, i\}, i \in C^K \setminus \{\mathbf{D}\}, \tag{S.13}$$

$$\sum_{i' \in C} \hat{x}_{i',5}^{(i)} = 1, \quad i \in C^K \setminus \{\mathbf{D}\}. \tag{S.14}$$

UNIFAC Model

The equations proposed by Smith et al.[5] are used as they are formulated in a convenient form for programming purposes. These equations are slightly modified in order to avoid numerical difficulties when evaluating activity coefficients.

Activity coefficient

$$\ln \gamma_{i,t}(x,T) = \ln \gamma^C_{i,t}(x,T) + \ln \gamma^R_{i,t}(x,T), \quad i \in C^K; t \in T \tag{S.15}$$

Combinatorial part of activity coefficient

$$\ln \gamma^C_{i,t}(x,T) = 1 - J_{i,t} + \ln J_{i,t} - 5q_{i,t}\left(1 - \frac{J_{i,t}}{L_{i,t}} + \ln \frac{J_{i,t}}{L_{i,t}}\right), \quad i \in C^K; t \in T \tag{S.16}$$

$$r_i = \sum_{k \in K} n_{i,k} R_k, \quad i \in C^K \tag{S.17}$$

$$q_i = \sum_{k \in K} n_{i,k} Q_k, \quad i \in C^K \tag{S.18}$$

$$r_s = \sum_{k \in K} n_{s,k} R_k, \quad s \in S \tag{S.19}$$

$$q_s = \sum_{k \in K} n_{s,k} Q_k, \quad s \in S \tag{S.20}$$

$$r_q = \sum_{s \in S} r_s y_{q,s}, \quad q \in Q; s \in S \tag{S.21}$$

$$q_q = \sum_{s \in S} q_s y_{q,s}, \quad q \in Q; s \in S \tag{S.22}$$

$$J_{i,t} = \frac{r_i}{\sum_{i \in C} r_i x_{i,t}}, \quad i \in C^K; t \in T \tag{S.23}$$

$$L_{i,t} = \frac{q_i}{\sum_{i \in C} q_i x_{i,t}}, \quad i \in C^K; t \in T \tag{S.24}$$

Residual part of activity coefficient

$$\ln \gamma^R_{i,t}(x,T) = q_i\left[1 - \sum_{k \in K}\left(\theta_{k,t}\frac{\beta_{i,k,t}}{\omega_{k,t}} - e_{i,k}\ln\frac{\beta_{i,k,t}}{\omega_{k,t}}\right)\right], \quad i \in C^K; t \in T \tag{S.25}$$

$$e_{i,k} = \frac{n_{i,k} Q_k}{q_i}, \quad i \in C^K; k \in K \tag{S.26}$$

$$\beta_{i,k,t} = \sum_{m \in K} \frac{n_{i,m} Q_m \psi_{m,k,t}}{q_i}, \quad i \in C^K; k \in K; t \in T \tag{S.27}$$

$$\theta_{k,t} = \frac{\sum_{i \in C} x_{i,t} n_{i,k} Q_k}{\sum_{i \in C} x_{i,t} q_i}, \quad k \in K; t \in T \tag{S.28}$$

$$\omega_{k,t} = \sum_{m \in K} \theta_{m,t} \psi_{m,k,t}, \quad k \in K; t \in T \tag{S.29}$$

$$\psi_{m,k,t} = \exp\left(\frac{-a_{m,k}}{T_t}\right), \quad m \in K; k \in K; t \in T \tag{S.30}$$

Solvent Lists

List I

1. 1,2-Propanediol
2. Acetic acid
3. Acetone
4. Diethoxymethane
5. Ethanol
6. Ethyl acetate
7. Formic acid
8. Isobutyl acetate
9. Isopropyl acetate
10. Isobutanol
11. Isopropanol
12. Methyl acetate

13. Methyl ethyl ketone (MEK)
14. Methyl tert-butyl ether (MTBE)
15. n-Butanol
16. 2-Butanol
17. tert-Amyl alcohol (t-AmOH)
18. t-Butanol
19. Triethyl amine
20. Water
21. Anisole
22. n-Butyl acetate
23. Trifluoroethanol
24. Trifluoroacetic acid
25. 1-Propanol
26. n-Pentane
27. Diethyl ether
28. Cyclohexanone
29. Ethyl formate
30. Nitrobenzene
31. Dimethyl sulfide
32. 2-Methyl pentane

33. 2-Pentanol

34. 2-Pentanone

35. 3-Pentanone

36. Cyclopentyl methyl ether

37. 2-2-4-Trimethyl pentane

38. Methyl cyclopentane

39. Propyl acetate

40. t-Amyl methyl ether

41. t-Butyl acetate

42. t-Butyl ethyl ether

List II

1. 1,2-Dimethoxyethane

2. 1,4-Dioxane

3. Acetic acid

4. Acetone

5. Acetonitrile

6. Chlorobenzene

7. Chloroform

8. Cyclohexane

9. DMAC
10. Ethanol
11. Ethyl acetate
12. Formic acid
13. Isopropyl acetate
14. Isobutanol
15. Isobutyl acetate
16. Isopropanol
17. Methyl acetate
18. MIBK
19. MEK
20. MTBE
21. n-Butanol
22. 2-Butanol
23. n-Hexane
24. Nitromethane
25. o-Xylene
26. t-AmOH
27. t-Butanol
28. Toluene

29. Triethyl amine
30. Water
31. Anisole
32. n-Butyl acetate
33. 1-Propanol
34. n-Pentane
35. Carbon tetrachloride
36. Diethyl ether
37. Ethyl formate
38. Nitrobenzene
39. 2-Methyl pentane
40. 2-Pentanone
41. 3-Pentanone
42. 2,2,4-Trimethyl pentane
43. Methyl cyclohexane
44. Methyl cyclopentane
45. Propyl acetate
46. t-Amyl methyl ether
47. t-Butyl acetate
48. t-Butyl ethyl ether

49. DMF

Data

Melting points, heats of melting, heat capacities and pure-component densities of solids involved in the Mefenamic Acid (MA) case study

Table S.1: Physical properties of solids involved in the MA case study.

Compound $i \in C^K$	Melting Point $T_{m,i}$ (K)	Heat of Melting $\Delta H_{m,i}$ (J/mol)	Liquid-Phase Molar Heat Capacity $C_{p_i}^{(L)}$ (J/mol-K)	ρ_i (g/mL)
DMA	276.70	14940	242.20	0.993
CBA	414.10	26290	241.22	1.197
MA	503.50	38700	447.20	1.203

Standard heat of reaction of MA synthesis

Table S.2: Standard heat of reaction of MA synthesis.

Reaction	Standard Heat of Reaction $\Delta H_R(T_R)$ (J/mol)
Synthesis of MA from DMA and CBA	-95067

Solvent Properties

Table S.3: Physical properties of solvent candidates in List II.

Solvent $s \in S$	Boiling Point $T_{b,s}$ (K)	Melting Point $T_{m,s}$ (K)	Molar Mass MW_s (g/mol)	Density ρ_s (g/mL)	Heat Capacity $C_{ps}^{(L)}$ (J/mol-K)	Heat of vaporisation ΔH_{V_s} (J/mol)
1-2-Dimethoxyethane	358.15	204.15	90.12	0.87	191.14	32420
1,4-Dioxane	374.15	285.15	88.11	1.03	149.65	37500
Acetic acid	391.00	289.00	60.05	1.05	123.10	51600
Acetone	329.20	178.50	58.08	0.79	125.45	29100
Acetonitrile	355.00	228.00	41.05	0.78	91.70	29750
Chlorobenzene	404.00	228.00	112.56	1.11	152.10	40100
Chloroform	334.40	209.70	119.38	1.49	114.25	31400
Cyclohexane	353.90	279.62	84.16	0.78	156.00	29970
DMAC	438.20	253.00	87.12	0.94	178.20	45100
Ethanol	351.40	159.00	46.07	0.79	112.40	38560
Ethyl acetate	350.20	189.60	88.11	0.90	168.94	31940
Formic acid	373.90	281.50	46.03	1.22	99.04	22690
Isobutyl acetate	399.20	195.00	116.16	0.87	240.20	39900
Isopropyl acetate	362.00	200.00	102.13	0.87	196.60	32900
Isobutanol	381.04	165.00	74.12	0.80	181.05	43290
Isopropanol	355.80	184.00	60.10	0.79	161.20	39850
Methyl acetate	330.00	175.00	74.08	0.93	141.34	30320
MIBK	390.00	188.50	100.16	0.80	211.90	40650
MEK	353.15	186.15	72.11	0.81	159.00	31300
MTBE	328.30	164.00	88.15	0.74	187.50	27940
n-Butanol	390.80	183.30	74.12	0.81	176.86	43290
2-Butanol	373.00	158.00	74.12	0.81	197.10	40750
n-Hexane	342.00	178.00	86.18	0.66	265.20	28850
Nitromethane	374.30	244.50	61.04	1.13	105.98	33990
o-Xylene	417.50	249.00	106.17	0.88	187.65	36240
t-AmOH	375.00	264.00	88.15	0.81	247.15	40110
t-Butanol	355.00	298.00	74.12	0.81	215.37	39070
Toluene	384.00	178.00	92.14	0.86	157.09	33180
Triethyl amine	362.00	158.45	101.19	0.73	216.43	31010
Water	373.15	273.15	18.02	0.99	75.38	40660
Anisole	427.00	236.00	108.14	0.99	199.00	46910
n-Butyl acetate	399.20	195.00	116.16	0.88	225.11	36280
1-Propanol	370.00	147.00	60.10	0.80	143.96	41440
n-Pentane	309.00	143.00	72.15	0.63	167.19	25790
Carbon tetrachloride	349.87	250.23	153.82	1.59	131.30	29820
Diethyl ether	307.80	256.80	74.12	0.71	172.50	26520
Ethyl formate	327.10	193.00	74.08	0.92	144.30	32110
Nitrobenzene	484.00	278.80	123.11	1.21	181.13	54500
2-Methyl pentane	334.00	120.00	86.18	0.66	194.19	27790
2-Pentanone	375.00	196.00	86.13	0.81	185.40	38460
3-Pentanone	375.00	234.20	86.13	0.82	196.40	33450
2,2,4-Trimethyl pentane	372.40	165.77	114.23	0.69	242.49	30790
Methyl cyclohexane	374.00	146.60	98.19	0.77	184.38	31270
Methyl cyclopentane	345.00	131.00	84.16	0.75	158.70	29080
Propyl acetate	374.70	178.15	102.13	0.88	194.10	33920
t-Amyl methyl ether	359.40	193.00	102.17	0.77	222.00	27940
t-Butyl acetate	369.80	211.15	116.16	0.86	231.00	36280
t-Butyl ethyl ether	346.00	179.15	102.17	0.74	218.00	29760
DMF	426.15	212.15	73.10	0.95	146.05	43600

Table S.4: Physical properties of additional solvent candidates in List I.

Solvent $s \in S$	Boiling Point $T_{b,s}$ (K)	Melting Point $T_{m,s}$ (K)	Molar Mass MW_s (g/mol)	Density ρ_s (g/mL)
1,2-Propanediol	460.15	213.15	76.10	1.04
Diethoxymethane	361.15	206.65	104.15	0.83
Trifluoroethanol	347.10	229.70	100.04	1.39
Trifluoroacetic acid	345.50	257.80	114.02	1.54
Cyclohexanone	420.80	226.00	98.14	0.95
Dimethyl sulfide	311.00	175.00	62.13	0.85
2-Pentanol	392.00	200.00	88.15	0.81
Cyclopentyl methyl ether	379.00	133.00	100.16	0.86

Multi-objective optimisation results

Table S.5: Pareto-optimal solutions of the MOO problem involving the SEF, Y_P and PP.

Solution	ϵ_2 (%)	ϵ_3 (%)	Solvents	SEF (kg/kg API)	Y_P (%)	PP (%)
1	81.80	95.70	s1: Anisole<br>s2: Acetic acid	3.84	84.90	95.70
2		96.68	s1: Anisole<br>s2: n-Butanol	3.93	87.12	96.68
3		97.65	s1: Nitrobenzene<br>s2: n-Butanol	4.29	87.14	97.65
4		98.63	s1: 2-Pentanol<br>s2: Anisole	5.16	86.81	98.63
5		99.60		Infeasible solution		
6	83.38	95.70	s1: 2-Pentanol<br>s2: Anisole	3.82	83.38	95.74
7		96.68	s1: Anisole<br>s2: Isobutyl acetate	4.00	87.82	96.68
8		97.65	s1: n-Butyl acetate<br>s2: Anisole	4.37	87.83	97.65
9		98.63	s1: 2-Pentanol<br>s2: Anisole	5.16	86.81	98.63
10		99.60		Infeasible solution		
11	84.95	95.70	s1: Anisole<br>s2: Isobutyl acetate	3.83	84.95	95.76
12		96.68	s1: Anisole<br>s2: 2-Pentanol	3.95	86.78	96.68
13		97.65	s1: Anisole<br>s2: n-Butanol	4.27	87.10	97.65
14		98.63	s1: Anisole<br>s2: Isobutyl acetate	5.26	87.84	98.63
15		99.60		Infeasible solution		
16	86.53	95.70	s1: Nitrobenzene<br>s2: Cyclohexanone	3.84	86.53	95.70
17		96.68	s1: Nitrobenzene<br>s2: n-Butanol	3.94	87.18	96.68
18		97.65	s1: Anisole<br>s2: n-Butanol	4.27	87.10	97.65
19		98.63	s1: 2-Pentanol<br>s2: Anisole	5.16	86.81	98.63
20		99.60		Infeasible solution		
21, 22, 23	88.10	95.70, 96.68, 97.65	s1: Nitrobenzene<br>s2: Cyclohexanone	5.29	88.10	98.56
24		98.63		Infeasible solution		
25		99.60		Infeasible solution		

Model equations that can be used in case $s1$ or $s2$ are identical to sw

In the following equations, $\tilde{M}_x$ and M_γ are sufficiently large numbers.

Solubility

Consider stream 12 with the mole fraction vector $\mathbf{x}_{12}$. Let us define the mole fractions of this stream as follows:

- $x_{s1,12}$ is the mole fraction of solvent $s1$ in stream 12, which is equal to either the value of $x_{s1,12}$ in the original formulation or zero, depending on whether or not its identity is the same of that of sw;
- $x_{s2,12}$ is the mole fraction of solvent $s2$ in stream 12, which is equal to either the value of $x_{s2,12}$ in the original formulation or zero, depending on whether or not its identity is the same of that of sw;
- $x_{swt,12}$ is the mole fraction of wash solvent sw in stream 12. Mathematically, this is equal to the sum of $x_{sw,12}$, $x_{s1+sw,12}$ and $x_{s2+sw,12}$ where $x_{q+sw,12}$ is the combined mole fraction of solvent $q \in Q$ and solvent sw in the case of matching solvent identities in stream 12. Note that $x_{sw,12}$ is set to zero in case the identity of either $s1$ or $s2$ matches sw, and $x_{q+sw,12}$ set to zero otherwise;
- x_i is the mole fraction of component $i \in C^K$.

Accordingly, $\mathbf{x}_{12} = (x_{swt,12}, x_{s1,12}, x_{s2,12}, x_i)$ for $i \in C^K$. Based on the above, the following equations can be used in the modified CAM^{b}D formulation:

$$-\tilde{M}_x(1-y_{q,sw}) \leq x_{q,12} \leq \tilde{M}_x(1-y_{q,sw}), \quad q \in Q, \tag{S.31}$$

$$-\tilde{M}_x(1-y_{q,sw}) \leq x_{q+sw,12} - (x_{q,12} + x_{sw,12}) \leq \tilde{M}_x(1-y_{q,sw}), \quad q \in Q, \tag{S.32}$$

$$-\tilde{M}_x y_{q,sw} \leq x_{q+sw,12} \leq \tilde{M}_x y_{q,sw}, \quad q \in Q, \tag{S.33}$$

$$-\tilde{M}_x(1-y_{q,sw}) \leq x_{sw,12} \leq \tilde{M}_x(1-y_{q,sw}), \quad q \in Q, \tag{S.34}$$

$$x_{swt,12} = x_{sw,12} + x_{s1+sw,12} + x_{s2+sw,12}. \tag{S.35}$$

where $y_{q,sw}$ is a binary variable which equals 1 in case the identity of sw from set S is assigned to q and 0 otherwise.

For example, assume the identity of $s2$ was the same as that of sw. It means that $y_{s2,sw} = 1$ and $y_{s1,sw} = 0$ (since $s1$ and $s2$ cannot have the same identity). It follows that:

$$x_{s2,12} = 0, \tag{S.36}$$

$$x_{sw,12} = 0, \tag{S.37}$$

$$x_{s1+sw,12} = 0, \tag{S.38}$$

$$x_{s2+sw,12} = x_{s2,12} + x_{sw,12}, \tag{S.39}$$

$$x_{swt,12} = 0 + 0 + x_{s2+sw,12} = x_{s2,12} + x_{sw,12}. \tag{S.40}$$

Hence, the mole fraction vector $\mathbf{x}_{12}$ is $\mathbf{x}_{12} = (x_{swt,12} = x_{s2,12} + x_{sw,12},\ x_{s1,12},\ x_{s2,12} = 0,\ x_i)$ for $i \in C^K$.

Miscibility

Miscibility calculations are performed using the modified mole fractions of stream 12 as derived in the previous section. The following constraints ensure that the miscibility between $s1$ and sw is checked only if $y_{s1,sw} = 0$:

$$-\tilde{M}_x y_{s1,sw} \leq \tilde{x}_{s1,12} - \frac{x_{s1,12}}{x_{s1,12} + x_{swt,12}} \leq \tilde{M}_x y_{s1,sw}, \tag{S.41}$$

$$\frac{\partial \ln \tilde{\gamma}_{s1,12}(\tilde{\mathbf{x}}_{12}^{s1}, T_{12})}{\partial \tilde{x}_{s1,12}} + \frac{1}{\tilde{x}_{s1,12}} \geq -M_\gamma y_{s1,sw}. \tag{S.42}$$

Similarly, the following constraints ensure that the miscibility between $s2$ and sw is checked only if $y_{s2,sw} = 0$:

$$-\tilde{M}_x y_{s2,sw} \leq \tilde{x}_{s2,12} - \frac{x_{s2,12}}{x_{s2,12} + x_{swt,12}} \leq \tilde{M}_x y_{s2,sw}, \tag{S.43}$$

$$\frac{\partial \ln \tilde{\gamma}_{s1,12}(\tilde{\mathbf{x}}_{12}^{s2}, T_{12})}{\partial \tilde{x}_{s2,12}} + \frac{1}{\tilde{x}_{s2,12}} \geq -M_\gamma y_{s2,sw}. \tag{S.44}$$

References

(1) Muhieddine, M. H.; Viswanath, S. K.; Armstrong, A.; Galindo, A.; Adjiman, C. S. Model-based solvent selection for the synthesis and crystallisation of pharmaceutical compounds. *Chem. Eng. Sci.* **2022**, 118125.

(2) Gmehling, J. G.; Anderson, T. F.; Prausnitz, J. M. Solid-liquid equilibria using UNIFAC. *Ind. Eng. Chem. Fundam.* **1978**, *17*, 269–273.

(3) Sandler, S. *Chemical and Engineering Thermodynamics, 3rd edn, Sec. 8.5*; J. Wiley & Sons Inc., New York, 1999.

(4) Fredenslund, A.; Jones, R. L.; Prausnitz, J. M. Group-contribution estimation of activity coefficients in nonideal liquid mixtures. *AIChE J.* **1975**, *21*, 1086–1099.

(5) Smith, J. M.; Van Ness, H. C.; Abbott, M. M. *Introduction to Chemical Engineering Thermodynamics*; McGraw-Hill, New York, 2005.
